# ETS Transcription Factors Control Transcription of EZH2 and Epigenetic Silencing of the Tumor Suppressor Gene Nkx3.1 in Prostate Cancer

**DOI:** 10.1371/journal.pone.0010547

**Published:** 2010-05-10

**Authors:** Paolo Kunderfranco, Maurizia Mello-Grand, Romina Cangemi, Stefania Pellini, Afua Mensah, Veronica Albertini, Anastasia Malek, Giovanna Chiorino, Carlo V. Catapano, Giuseppina M. Carbone

**Affiliations:** 1 Laboratory of Experimental Oncology, Oncology Institute of Southern Switzerland, Bellinzona, Switzerland; 2 Laboratory of Cancer Genomics, Fondo Edo Tempia, Biella, Italy; Baylor College of Medicine, United States of America

## Abstract

**Background:**

ETS transcription factors regulate important signaling pathways involved in cell differentiation and development in many tissues and have emerged as important players in prostate cancer. However, the biological impact of ETS factors in prostate tumorigenesis is still debated.

**Methodology/Principal Findings:**

We performed an analysis of the ETS gene family using microarray data and real-time PCR in normal and tumor tissues along with functional studies in normal and cancer cell lines to understand the impact in prostate tumorigenesis and identify key targets of these transcription factors. We found frequent dysregulation of ETS genes with oncogenic (i.e., ERG and ESE1) and tumor suppressor (i.e., ESE3) properties in prostate tumors compared to normal prostate. Tumor subgroups (i.e., ERG^high^, ESE1^high^, ESE3^low^ and NoETS tumors) were identified on the basis of their ETS expression status and showed distinct transcriptional and biological features. ERG^high^ and ESE3^low^ tumors had the most robust gene signatures with both distinct and overlapping features. Integrating genomic data with functional studies in multiple cell lines, we demonstrated that ERG and ESE3 controlled in opposite direction transcription of the Polycomb Group protein EZH2, a key gene in development, differentiation, stem cell biology and tumorigenesis. We further demonstrated that the prostate-specific tumor suppressor gene Nkx3.1 was controlled by ERG and ESE3 both directly and through induction of EZH2.

**Conclusions/Significance:**

These findings provide new insights into the role of the ETS transcriptional network in prostate tumorigenesis and uncover previously unrecognized links between aberrant expression of ETS factors, deregulation of epigenetic effectors and silencing of tumor suppressor genes. The link between aberrant ETS activity and epigenetic gene silencing may be relevant for the clinical management of prostate cancer and design of new therapeutic strategies.

## Introduction

Cancer of the prostate is the most common cancer and a leading cause of cancer death in western countries [1]. Prostate cancer has a highly heterogeneous clinical behavior and little is known about the molecular mechanisms contributing to this heterogeneity [1]. Recently, ETS transcription factors have emerged as important elements in prostate tumorigenesis due to the finding of recurrent translocations involving ETS genes, the most frequent being the TMPRSS2: ERGa gene fusion leading to over-expression of full length ERG [2], [3], [4]. However, the biological impact of translocated ETS genes is still debated. Recent reports suggest that ERG over-expression is not sufficient to induce neoplastic transformation and cooperation with other oncogenic pathways, such as PTEN loss and PI3K/AKT dysregulation, is necessary [5], [6], [7], [8], [9]. The human ETS family includes 27 members that share a highly conserved DNA binding domain and are nodal points of various signaling pathways controlling cell proliferation, differentiation and survival [10]. Although there is great potential for overlap, individual ETS factors have distinct features that manifest through positive and negative regulation of different subsets of genes and biological processes [10]. Moreover, in many tissues ETS factors constitute complex regulatory networks with specific cellular responses depending on the balance between factors with similar or opposite functions [10]. Most ETS factors, like those translocated in prostate cancer, promote cell proliferation, survival and transformation, while others act as tumor suppressors [10]. Recently, we found that the epithelial-specific ETS factor ESE3 was frequently down-regulated in prostate cancer, negatively affected cell proliferation and survival, and acted as tumor suppressor in prostate epithelial cells [11]. Thus, to understand the overall impact of ETS gene deregulation in tumorigenesis and identify key targets of deregulated ETS factors, it would be important to consider the entire set of ETS genes expressed in a given tissue.

In this study, through a comprehensive analysis of the ETS gene family in prostatic normal and tumor tissues, we identified tumor subgroups with distinct ETS expression patterns. Besides already known ETS targets, we discovered previously unrecognized genes and pathways linked to aberrant ETS activity. By integrating genomic data with functional studies, we established that the Polycomb Group (PcG) protein EZH2 is a direct target of ERG and ESE3, and a key player in transcriptional silencing of the prostate specific tumor suppressor gene Nkx3.1. Taken together, our data reveal more frequent and complex alterations of ETS genes than previously recognized and identify key genes like EZH2 and Nkx3.1 contributing to the reprogramming of prostate epithelial cell transcriptome in response to aberrant expression of oncogenic and tumor suppressor ETS factors. These findings may be relevant for the clinical management for prostate cancer and design of new therapeutic strategies.

## Results

### ETS Gene Expression Patterns Define Prostate Cancer Subgroups

To gain a comprehensive view of the ETS transcriptional network in prostate cancer, we examined the expression of the ETS gene family in microarray datasets from primary prostate cancer (*n = 59*) and normal prostate (*n = 14*) clinical samples. Analysis of microarray data showed that several ETS genes were differentially expressed in tumor samples compared to normal prostate. Quantitative RT-PCR (QRT-PCR) was used to confirm the differential expression of the most frequently altered ETS factors (Fig. S1). The most significantly affected ETS genes were ERG, ESE3 and the epithelial-specific ETS factor-1 (ESE1/ELF3/ESX). We previously showed that ESE3, that is expressed in normal prostate epithelial cells, negatively affected proliferation and survival of prostate cancer cells and proposed that it acted as a tumor suppressor [11]. ESE1, which is closely related to ESE3, is expressed in normal epithelial cells of various organs, including prostate, breast and lung and is known to act as an oncogene when over-expressed in breast epithelial cells [12], [13]. However, up-regulation of ESE1 in prostate tumors has not been reported before. Overall, dysregulated expression of ETS genes was very frequent in prostate tumors. Indeed, most of the tumors had at least one up- or down- regulated ETS gene compared to normal prostate and often multiple ETS were simultaneously affected.

**Figure 1:**
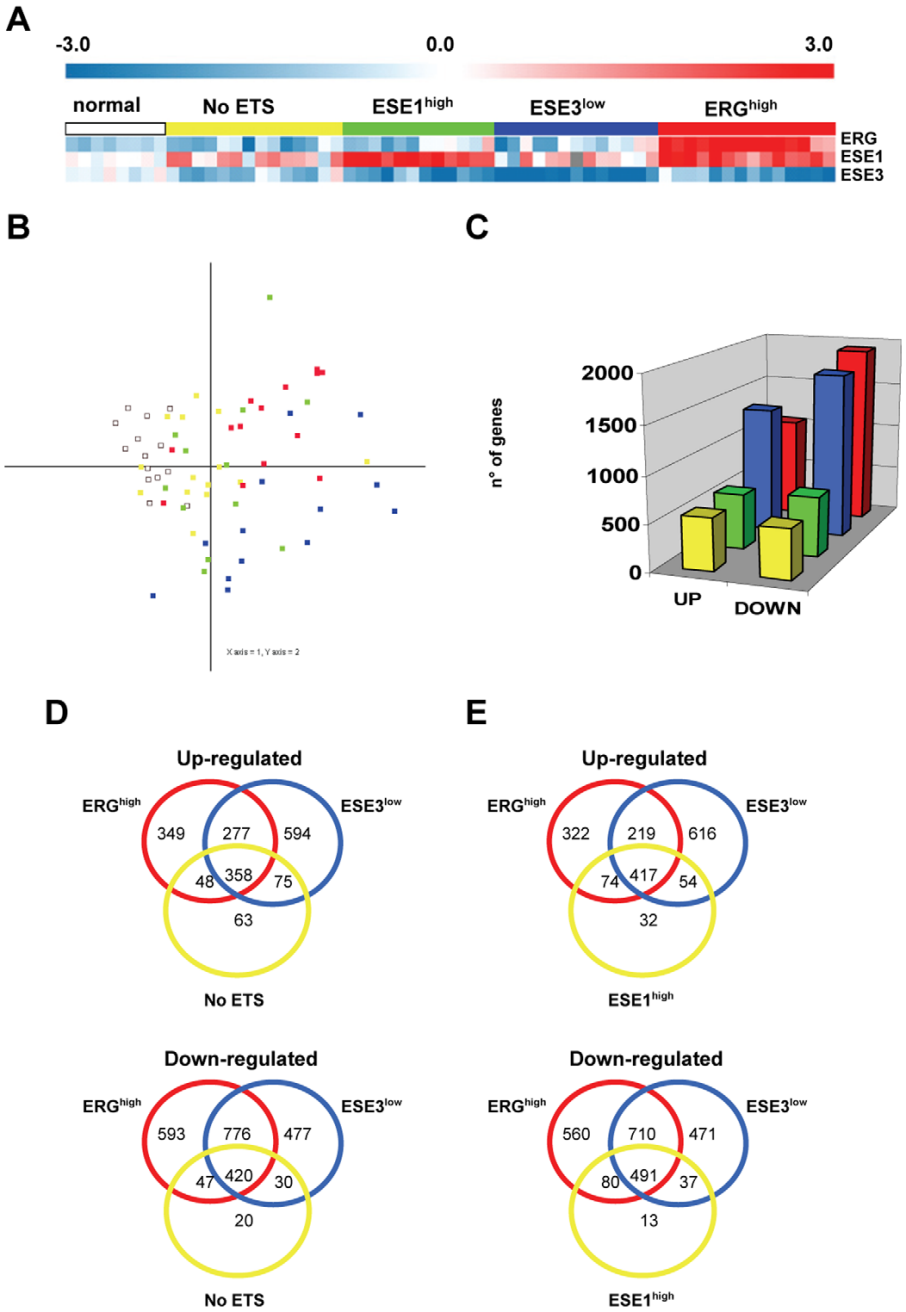
ETS gene signatures in prostate cancer. **A.** Expression of ERG, ESE1 and ESE3 determined by qRT-PCR in normal prostate and prostate tumors. Tumors are grouped according to the predominantly expressed ETS factor. **B.** Principal component analysis. Dots represent individual samples with their location determined by the principal components of the transcriptome. **C.** Number of differentially expressed genes with Q≤0.1 in each ETS subgroup. **D-E.** Venn diagrams showing shared and distinct differentially expressed genes among the indicated tumor subgroups.

Our objective was to understand how these individual and compounded ETS alterations could affect the biology of prostate cancer. Using QRT-PCR data on ETS gene expression and genomic data we evaluated whether specific transcriptional profiles were associated with the distinct ETS expression patterns. We used several criteria to minimize the potential confounding effects of the presence of multiple ETS factors. First, QRT-PCR data were used to classify accurately tumors according to their ETS gene expression status. Then, only tumors with very high or very low expression of a given ETS (i.e., ≥4-fold higher or lower than the average value in normal prostate) were assigned to a group and included in the analysis. Using these criteria approximately 80% of prostate cancers had highly deregulated expression of at least one predominant ETS gene. On these bases, we identified three major tumor subgroups characterized by the predominant dysregulation of an ETS factor: i) tumors with high ERG expression (ERG^high^, *n = 14*), ii) tumors with high ESE-1 expression (ESE1^high^, *n = 12*) and iii) tumors with low ESE3 expression (ESE3^low^, *n = 13*). A fourth group (NoETS, *n = 14*) included tumors that had normal-like levels of all ETS gene (Fig. 1A). Eight tumors with ≥4-fold over-expression either of ETV1, ETV4, ETS2 or ETS1 were excluded from the analysis because of their limited numbers. ESE1 was highly expressed in 26 of the 59 (44%) prostate tumors, but only in 12, which were included in the ESE1^high^ expressing group, it was the only over-expressed ETS. ESE3 was down-regulated ≥4 fold in 27 of the 59 (46%) prostate tumors but only in the 13 cases, which were included in the ESE3^low^ expressing group, it was the only deregulated ETS. We applied a similar approach to a publicly available microarray dataset from an independent study [14] obtaining a similar distribution of prostate tumors in four major subgroups (Fig. S2). Interestingly, in our series 6 and 8 of the 14 ERG^high^ tumors had concomitantly dysregulated expression of ESE3 and ESE1, respectively (Fig. 1A). Similarly in the public dataset the 15 ERG^high^ tumors had concomitantly dysregulated expression of ESE3 and ESE1 (Fig. S2A). Thus, ERG over-expression could clearly coexist with dysregulated expression of these other ETS factors. In our tumor series, 11 of the 14 ERG^high^ tumors (79%) were positive for the TMPRSS2: ERGa fusion transcript assessed by end-point RT-PCR (Fig. S3A). The other ERG over-expressing tumors had likely other types of fusion transcripts not detected by the assay. Seven tumors had very low levels of TMPRSS2: ERGa transcript by end-point RT-PCR, normal-like expression of ERG by QRT-PCR and were not included in the ERG^high^ group. None of the 8 normal samples and of the 11 benign prostatic hyperplasia (BPH) samples examined had evidence of the TMPRSS2: ERGa transcript (Fig. S3A). Clinical and pathological parameters of the four tumor subgroups are shown in Fig. S3B. There was no statistically significant association between ETS subgroups and any of the assessed clinical/pathological parameters.

### Transcriptional Programs in Prostate Cancer Subgroups

We used Principal Component Analysis (PCA) to assess the global degree of similarity or divergence of the individual transcriptomes of normal and prostate cancer samples. As shown in Fig. 1B, tumors belonging to different subgroups formed partially distinct clusters suggesting that divergence in the transcriptional programs depended at least in part on their ETS gene expression patterns. ERG^high^ and ESE3^low^ tumors were the most distant from normal prostate and largely distinct from the other subgroups. Next, we compared the transcriptional profiles of the tumor subgroups with that of normal prostate using Gene Expression Profile Analysis Suite (GEPAS) [15] to identify common and distinct features and extract ETS-specific gene signatures. The number of differentially expressed genes (Q≤0.1) in each subgroup is shown in Fig. 1C and the gene lists are shown in Table S1. ERG^high^ and ESE3^low^ tumors had the most robust gene signatures with the largest number of differentially expressed genes relative to normal prostate, while the number of differentially expressed genes was considerably less in ESE1^high^ and NoETS tumors.

Next, we crossed the lists of differentially expressed genes relative to normal prostate to determine the degree of overlap and divergence among tumor subgroups (Fig. 1D-E). Notably, ERG^high^ and ESE3^low^ tumors shared many differentially expressed genes with a large overlap among both up-regulated and down- regulated genes, indicating that altered expression of ERG and ESE3 had partially similar effects. On the other hand, ERG^high^ and ESE3^low^ tumors had a large number of distinctive features relative to No ETS (Fig. 1D) and ESE1^high^ (Fig. 1E) tumors. These differences in gene set overlap were statistically significant (P<0.0001, Fisher Exact test). The genes modulated both in ERG^high^ and ESE3^low^ tumors but not in the other tumor subgroups, identified through a 3-way (Fig. 1D) and 4-way (Fig. S4) Venn diagrams, are listed in Table S2.

**Figure 2:**
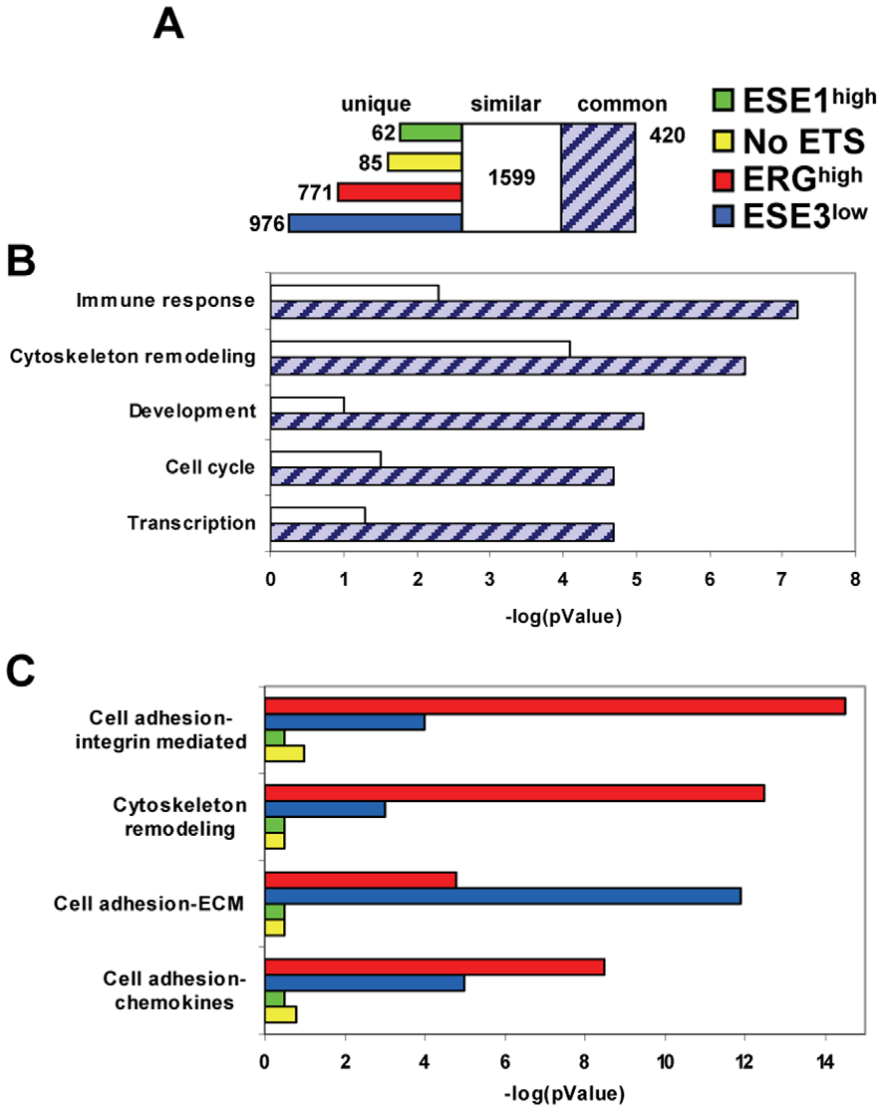
Functional analysis of the transcriptional programs of prostate tumor subgroups. **A**. Number of unique, similar and common features among the differentially expressed genes compared to normal prostate in ERG^high^, ESE3^low^, ESE1^high^ and NoETS tumors according to Metacore. **B.** Commonly affected GeneGo Pathway Maps in the tumor subgroups. **C.** Differentially affected GeneGo Pathway Maps in ERG^high^, ESE3^low^, ESE1^high^ and NoETS tumors.

To understand the functional implications of the differences among the tumor subgroups we used Metacore, a software suite for integrated functional analysis of the gene expression data [16]. Metacore allowed to map common, similar and unique features among tumor subgroups and define commonly and differentially affected Gene Ontology pathways. As shown in Fig. 2A, there were many common and similar features among the tumor subgroups. On the other hand, ERG^high^ and ESE3^low^ tumors had the largest number of unique features. The top commonly affected GeneGo pathway maps (GGPM), shown in Fig. 2B, included *immune response, cytoskeleton remodeling, development, cell cycle* and *transcriptional regulation*. They may represent genes and pathways commonly activated in tumors compared to normal tissue. The differentially affected GGPMs were prevalently or exclusively enriched in ERG^high^ and ESE3^low^ tumors (Fig. 2C). The top differentially affected GGMPs included *cell adhesion-integrin mediated*, *cytoskeleton remodeling*, *cell adhesion-ECM remodeling* and *cell adhesion-chemokines*, suggesting that activation of these pathways, related to cell migration and invasion, could be predominant features of these tumors. Interestingly, these data implicated also that ESE3 loss had consequences quite similar to ERG over-expression on the transcriptional program of prostate tumors.

### ERG Upregulates EZH2 Expression in Prostate Tumors

**Figure 3:**
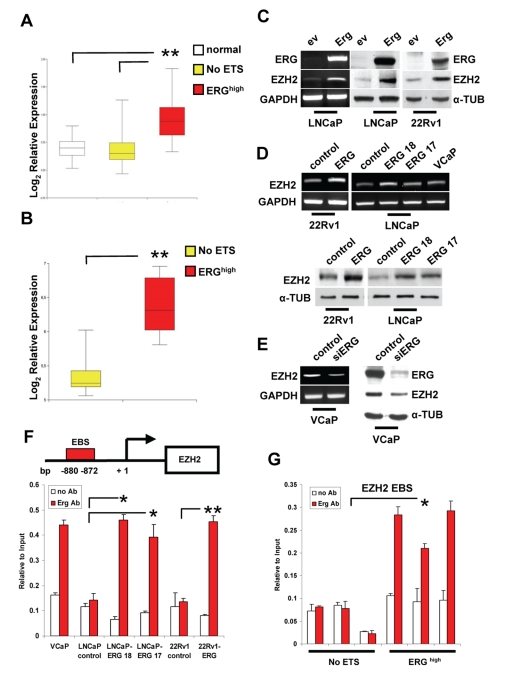
ERG induces EZH2 expression. **A.** EZH2 expression in tissue samples. Microarray data are presented as log2 ratios compared to the reference. **B.** EZH2 expression in NoETS and ERG^high^ tumors in the Wallace et al. microarray dataset. **C.** EZH2 expression in LNCaP and 22Rv1 cells transiently transfected with empty (*ev*) or ERG expression (*Erg*) vector determined by RT-PCR (*left*) and Western blot (*right*). **D.** EZH2 level in control and stable ERG-expressing 22Rv1 and LNCaP cells determined by RT-PCR (*upper*) and Western blot (*bottom*). **E.** EZH2 level in VCaP cells transiently transfected with control and ERG-specific (siERG) siRNA determined by RT-PCR (*left*) and Western blot (*right*). **F.** ChIP in the indicated cell lines with ERG antibody and qPCR with primer sets encompassing the EBS in the EZH2 promoter. Positive (MMP3 promoter) and negative (ETS2) controls are shown in Fig. S6A-B and 7A, respectively. **G** Tissue specimens of ERG^high^ and NoETS tumors were subjected to ChIP with anti-ERG antibody and analyzed by qPCR. ETS2 was used as negative control (Fig. S7B). *, P<0.01; **, P<0.0001.

Comprehensive evaluation of ETS gene expression and genomic data showed robust and partially overlapping gene signatures in the ERG^high^ and ESE3^low^ tumors with many activated and repressed genes. Next, we searched ERG^high^ and ESE3^low^ signatures for target genes that could act as key nodes mediating the effects of these aberrantly expressed ETS factors on the prostate cancer transcriptome. EZH2 was among the 169 up-regulated genes both in ERG^high^ and ESE3^low^ tumors genes (Fig. S4 and Table S2) while it was not increased in the other tumor subgroups. EZH2 was also positively correlated with ERG and negatively correlated with ESE3 in a correlation analysis of the entire microarray dataset (Table S3). EZH2 is a histone H3 lysine 27 (H3K27) methyltransferase and a key element in epigenetic gene silencing [17], [18]. H3K27 methylation is a histone mark that creates an anchoring point for the recruitment of additional chromatin remodeling factors inducing a repressive chromatin state [17]. Thus, the induction of EZH2 associated with ERG gain and ESE3 loss could contribute to the broad repressive signature observed in these tumors (Fig. 1C). EZH2 has been shown to be up-regulated in prostate cancers compared to normal prostate with particularly higher levels in high grade and metastatic tumors [19]. However, few factors have been identified that might increase EZH2 expression in prostate tumors [20], [21], [22], [23]. We found that EZH2 was significantly up-regulated in ERG^high^ tumors compared to both normal prostate and NoETS tumors (Fig. 3A), indicating that there might be a direct link between EZH2 and ERG expression that had not been recognized before. Consistent with this finding, EZH2 was significantly higher in ERG^high^ compared to NoETS tumors (Q<0.0001) also in an independent dataset (Fig. 3B).

Next, we probed the functional relationship between ERG and EZH2 and the possibility of direct regulation of EZH2 by ERG in prostate cancer cells, in which we experimentally up- and down-regulated ERG. In these experiments we over-expressed ERG in ERG-translocation negative 22Rv1 and LNCaP prostate cancer cells that do not express endogenous ERG. Upon transfection, 22Rv1 and LNCaP cells expressed ERG to levels similar to TMPRSS2: ERG translocation positive VCaP cells (Fig. S5A). In parallel, knock-down of ERG was performed in VCaP cells using an ERG specific siRNA. The level of ERG RNA and protein was significantly reduced in siRNA transfected cells compared to control VCaP cells (Fig. S5B). To validate these cellular models, we examined the expression of genes, like MMP3, PLA-1 and CRISP3, which were at the top of the list of up-regulated genes in ERG^high^ tumors and had been previously shown to be controlled by ERG in other cell systems [6]. Expression of these genes increased upon ERG over-expression in 22Rv1 and LNCaP cells (Fig. S5C) and decreased upon ERG knock-down in VCaP cells (Fig. S5D). These results demonstrated the adequacy of our cellular models to investigate potential ERG target genes along with the predictive value of the gene signatures that we derived from prostate cancer clinical samples.

Both transient and stable expression of ERG in ERG-negative LNCaP and 22Rv1 cells led to increased level of EZH2 (Fig. 3C-D). Furthermore, EZH2 mRNA and protein were reduced upon siRNA-mediated knock-down of ERG in VCaP cells (Fig. 3E). The changes in EZH2 expression observed upon up- and down- regulation of ERG suggested the possibility of direct regulation by ERG. We identified putative ETS binding sites (EBSs) within 1 kb of the EZH2 TSS by computational analysis and performed ChIP experiments to determine whether ERG was able to bind to these sites (Fig. 3F). Binding of ERG to the EZH2 promoter was observed in ERG translocation positive VCaP cells and in LNCaP and 22Rv1 cells upon stable expression of ERG, while no binding was seen in non-ERG expressing parental LNCaP and 22Rv1 cells (Fig. 3F). Similarly, ERG was bound to the MMP3 gene, a known ERG target used here as positive control, only in ERG expressing clones and in VCaP cells (Fig. S6A). No binding of ERG was seen to the ETS2 promoter, which was used as a negative control (Fig. S7A). The region of the ETS2 promoter assessed by ChIP included the transcription start site and both RNA polymerase II and Sp1 had been shown to bind to this region in gel shift and ChIP assays ([24] and data not shown). Taken together, these data supported the conclusion that ERG acted as a transcriptional activator of EZH2 by binding to its promoter. Finally, to prove that this interaction occurred also in clinical tumor specimens we performed ChIP to assess binding of ERG to the EZH2 promoter in ERG^high^ and NoETS samples (Fig. 3G). ERG was associated to the EZH2 promoter in ERG^high^ tumors while it was absent in NoETS tumors, consistent with the hypothesis that it controlled transcription of this gene. Specificity was demonstrated by the absence of binding of ERG to the ETS2 promoter in the tumor samples (Fig. S7B).

### ERG Represses Nkx3.1 in Prostate Tumors through EZH2 and Histone H3K27 Methylation

**Figure 4:**
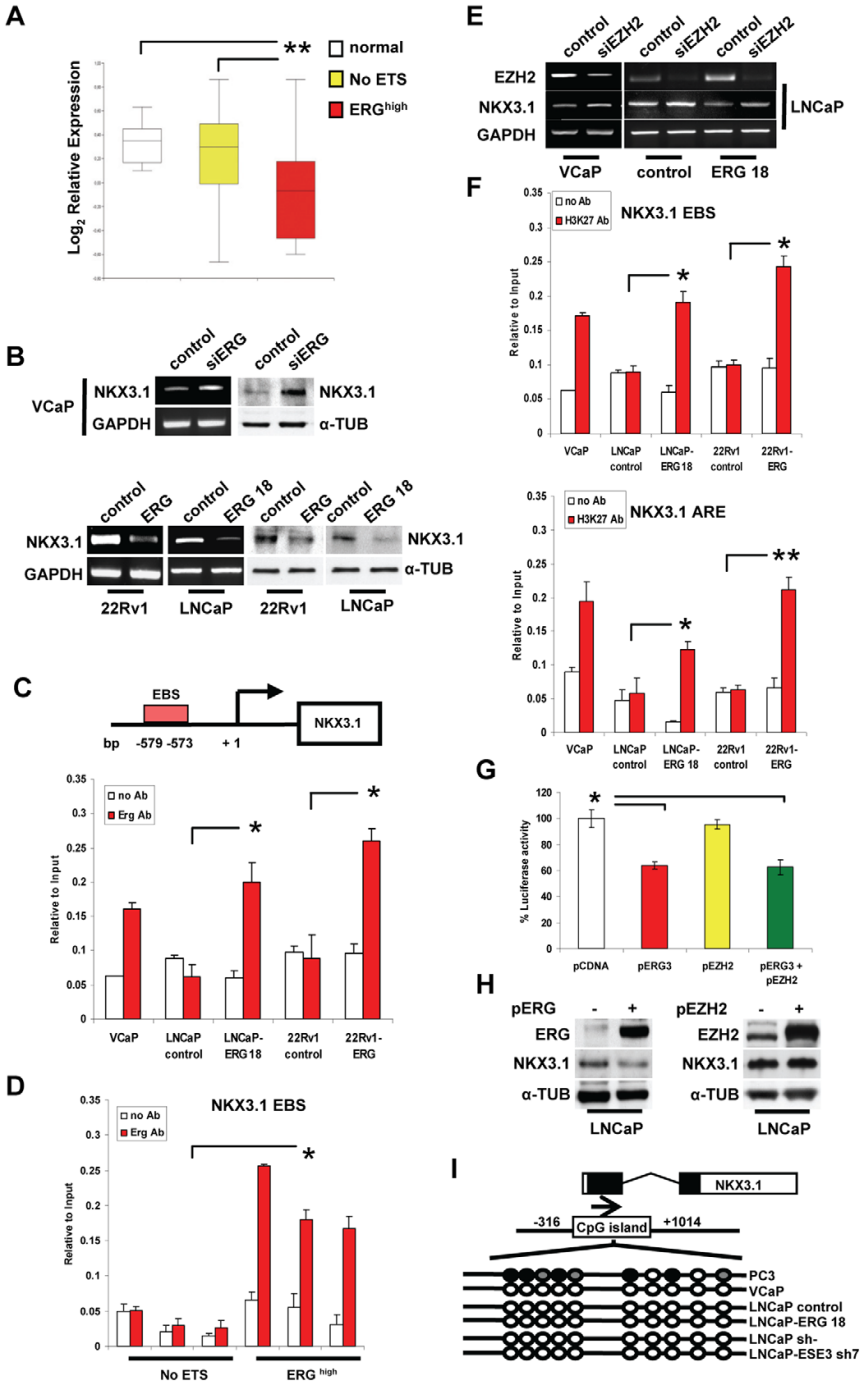
ERG represses Nkx3.1 expression. **A.** Nkx3.1 expression in normal and tumor tissue samples. **B.** Nkx3.1 level in LNCaP cells transiently transfected with empty (*ev*) or ERG expression (*Erg*) vector determined by Western blot (upper panel), Nkx3.1 level in VCaP cells transiently transfected with siERG and control siRNA determined by RT-PCR and Western blot (middle panel), Nkx3.1 level in control and stable ERG-expressing 22Rv1 and LNCaP cells analyzed by RT-PCR and Western blot (bottom panel). **C.** Binding of ERG to the Nkx3.1 promoter determined by ChIP and qPCR in indicated cell lines. Negative controls are shown in Fig. S7A. **D.** Tissue specimens of ERG^high^ and NoETS tumor were subjected to ChIP with anti-ERG antibody and analyzed by qPCR. Negative controls are shown in Fig. S7B. **E.** VCaP, parental (control) and ERG expressing (ERG 18) LNCaP cells transfected with EZH2 specific (siEZH2) and control siRNA and analyzed by RT-PCR**. F.** ChIP with an antibody for methylated H3K27 and qPCR with primer sets encompassing the EBS (*upper panel*) and an androgen responsive enhancer (ARE) (*bottom panel*) in the Nkx3.1 gene. ETS2 was used as negative control (Fig. S8A). **G.** Nkx3.1 promoter activity in LNCaP cells transfected with human Nkx3.1 promoter reporter along with the indicated expression vectors. Luciferase reporter activity was measured after 24 h. **H.** Nkx3.1 protein level in LNCaP cells transiently transfected with empty (−) or either ERG (pERG) or EZH2 (pEZH2) expression vector determined by Western blot. **I.** Nkx3.1 promoter methylation was assessed by bisulfite-treated DNA sequencing. Empty and filled circles represent unmethylated and methylated CpG sites, respectively**.** *, p<0.01; **, p<0.001.

In addition to up-regulated genes, the transcriptome of ERG^high^ tumors included numerous genes whose expression was significantly reduced compared to normal prostate, suggesting that these genes might be repressed either directly or indirectly by ERG. The list of down-regulated genes in ERG^high^ tumors included many relevant genes that could have significant impact on the prostate cancer biology. Among genes with known tumor suppressor functions, we focused on Nkx3.1, which was similarly affected in ERG^high^ and ESE3^low^ tumors. Nkx3.1 is a prostate-specific homeobox gene and a transcription factor that has critical functions in prostate development and tumor suppression [25]. Loss of Nkx3.1 expression is a frequent event in prostate tumorigenesis and has been attributed to various mechanisms including allelic loss, methylation and post-transcriptional silencing [25], [26], [27], [28]. We found that the level of Nkx3.1 was significantly reduced in ERG^high^ tumors compared to normal prostate and NoETS tumors (Fig. 4A). To determine whether Nkx3.1 down-regulation was functionally linked to ERG over-expression, we examined the level of Nkx3.1 in prostate cancer cells upon modulation of ERG. ERG knock-down in VCaP cells resulted in increased Nkx3.1 expression at the mRNA and protein level (Fig. 4B). On the other hand, Nkx3.1 level was reduced by stable expression of ERG in ERG negative LNCaP and 22RV1 cells, providing further evidence of control of Nkx3.1 expression by ERG (Fig. 4B). Since ETS factors can act as transcriptional activators or repressors depending on the promoter context [10], [29], we searched the Nkx3.1 promoter for possible EBS that could mediated ERG binding. Computational analysis showed the presence of a putative EBS in the Nkx3.1 promoter. ChIP assays showed binding of ERG to this region of the promoter in VCaP cells (Fig. 4C). ERG occupied the Nkx3.1 promoter also in LNCaP and 22Rv1 cells upon stable ERG expression and concomitantly to the silencing of the gene (Fig. 4C). Thus, binding of ERG to the Nkx3.1 promoter was associated with transcriptional repression of the gene. We observed Nkx3.1 promoter occupancy by ERG also in clinical tumor samples by performing ChIP in ERG^high^ and NoETS tumors. ERG was bound to the Nkx3.1 promoter in ERG^high^ tumors, consistent with the hypothesis that it controlled negatively transcription of the gene in this tumor subgroup (Fig. 4D).

To determine whether the induction of EZH2 by ERG could contribute to the silencing of Nkx3.1, we knocked-down EZH2 in ERG expressing cells. siRNA-mediated knock-down of EZH2 in VCaP cells increased expression of Nkx3.1, consistent with the hypothesis that the gene was under the control of EZH2 in these cells (Fig. 4E). Furthermore, EZH2 knock-down in ERG expressing LNCaP clones partially restored Nkx3.1 expression to a level similar to that of parental LNCaP cells (Fig. 4E). To further prove the role of EZH2, we determined by ChIP whether the Nkx3.1 promoter acquired the H3K27 methylation mark characteristic of EZH2 activity in cells in which the gene was repressed. We observed increased H3K27 methylation in the region surrounding the EBS, which we indentified in the promoter, and at the level of an androgen responsive enhancer (ARE), which is an important regulatory site in the Nkx3.1 gene [30] in ERG-translocation positive VCaP cells (Fig. 4F). A similar enrichment of H3K27 methylation was also observed in LNCaP and 22Rv1 cells with stable expression of ERG (Fig. 4F). Thus, Nkx3.1 acquired a repressive mark characteristic of EZH2 activity in an ERG-dependent manner. Taken together, these data established that Nkx3.1 was a target of both ERG and EZH2. ERG repressed Nkx3.1 directly by binding to its promoter and indirectly via the induction of EZH2 and H3K27 methylation. Luciferase reporter assays and transient transfection experiments supported this hypothesis. Nkx3.1 promoter activity and protein level were reduced upon transient expression of ERG in LNCaP cells (Fig. 4G-H). In contrast, transient transfection of EZH2 alone had no effect on Nkx3.1 promoter activity in reporter assay or Nkx3.1 protein level (Fig. 4G-H), indicating that EZH2 required ERG and stable expression to silence Nkx3.1. These findings are thus consistent with the ability of ETS factors to act alternatively as transcriptional activator and repressor [10], [29] and with the hypothesis that transcription factors can influence the recruitment of epigenetic effectors like EZH2 to gene promoters [31].

Acquisition of repressive histone marks, like H3K27 methylation, is only one of multiple mechanisms contributing to silencing of tumor suppressor genes in cancer cells [18]. The promoter of Nkx3.1 gene contains a CpG island and the gene has been reported to be silenced by CpG promoter methylation in prostate tumors [26]. Thus, we determined whether DNA methylation was also involved in ERG induced Nkx3.1 silencing. Bisulfite sequencing showed the absence of CpG methylation in the Nkx3.1 promoter in ERG-translocation positive VCaP cells and ERG expressing and non-expressing LNCaP cells (Fig. 4I). In contrast, the Nkx3.1 promoter was methylated in ERG-translocation negative PC3 prostate cancer cells that do not express Nkx3.1 (Fig. 4I) indicating that in these cells Nkx3.1 was silenced by CpG promoter methylation. Thus, ERG-induced Nkx3.1 silencing relied mainly on EZH2 and was independent of promoter methylation, consistent with reactivation of Nkx3.1 expression upon EZH2 knock-down.

### ESE-3 Represses EZH2 and Activates Nkx3.1 Transcription

**Figure 5:**
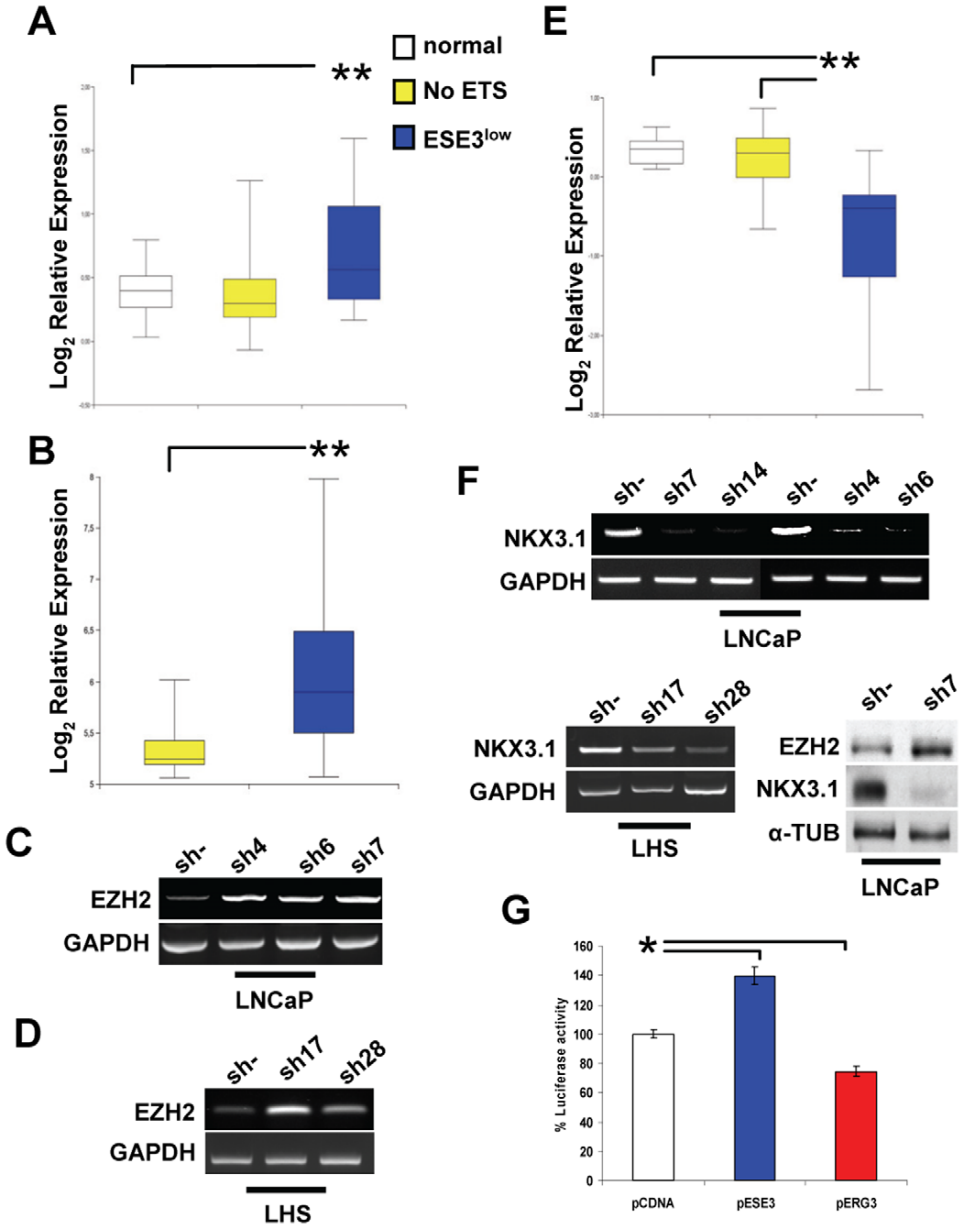
ESE-3 regulates EZH2 and Nkx3.1 expression. **A.** EZH2 level in normal prostate, ESE3^low^ and NoETS tumors. **B.** EZH2 level in NoETS and ESE3^low^ tumors in Wallace et al. microarray dataset. **C.** EZH2 level in control (sh-) and stable ESE3 knock-down (sh 4, 6, 7) LNCaP clones determined by RT-PCR. **D.** EZH2 level in control (sh-) and ESE3 knock-down (sh17, 28) LHS clones determined by RT-PCR. **E.** Nkx3.1 level in normal prostate, ESE3^low^ and NoETS tumors. **F.** Nkx3.1 level in control (sh-) and ESE3-knock-down LNCaP and LHS cells determined by RT-PCR and Western blot (*right bottom*). **G.** Nkx3.1 promoter activity in PC3 cells transfected with human NKx3.1 promoter reporter along with the indicated expression vectors. Luciferase reporter activity was measured after 24 h. *p<0.01; **, p<0.001.

The transcriptome of ERG^high^ and ESE3^low^ tumors shared many genes in common. This suggested that ERG and ESE3 could affect transcription of many genes in opposite directions and that ERG up-regulation and ESE3 down-regulation could results in partially similar effects on the prostate cancer transcriptome. As seen in ERG^high^ tumors, EZH2 was significantly higher in ESE3^low^ tumors compared to normal prostate and NoETS tumors (Fig. 5A). The inverse relationship between ESE3 and EZH2 expression level was also seen in an independent microarray dataset (Q<0.0004) [14] (Fig. 5B; S2D). These observations suggested that ESE3 could negatively regulate EZH2 in the prostate and loss of ESE3 could result in increased EZH2 expression in prostate tumors. To test this hypothesis, we generated clones of LNCaP cells (ESE3-*kd*) in which we stably knocked-down ESE3 using shRNA constructs. These clones had significantly lower levels of ESE3 compared to parental LNCaP cells, which express detectable levels of ESE3 (Fig. S5E). Consistent with our hypothesis, ESE3-*kd* LNCaP cells had higher expression of EZH2 than parental cells (Fig. 5C). To determine whether ESE3 controlled the expression of EZH2 also in normal prostate epithelial cells we stably knock-down ESE3 in immortalized LHS cells [32], which we have shown previously to express ESE3 [11]. shRNA-mediated knock-down was effective in reducing ESE3 level in LHS cells (Fig. S5E, *right panel*) and, consistent with our hypothesis, ESE3-*kd* LHS cells had a higher level of EZH2 than parental cells (Fig. 5D). Thus, experimentally reducing the level of ESE3 led to increased expression of EZH2, suggesting that ESE3 maintains EZH2 repressed in prostate epithelial cells.

Analysis of ESE3^low^ gene signature showed that, similar to ERG^high^ tumors, ESE3^low^ tumors had also lower levels of Nkx3.1 compared to normal prostate and NoETS tumors (Fig. 5E). Thus, ESE3 could control positively Nkx3.1. Consistently, we found that the level of Nkx3.1 was reduced upon ESE3 knock-down in LNCaP and LHS cells (Fig. 5F). Luciferase reporter assay showed that ESE3 increased Nkx3.1 promoter activity when transfected in ESE3 negative PC3 cells. On the other hand, ERG reduced luciferase activity confirming the opposing effects of these ETS factors on the Nkx3.1 promoter (Fig. 5G).

**Figure 6:**
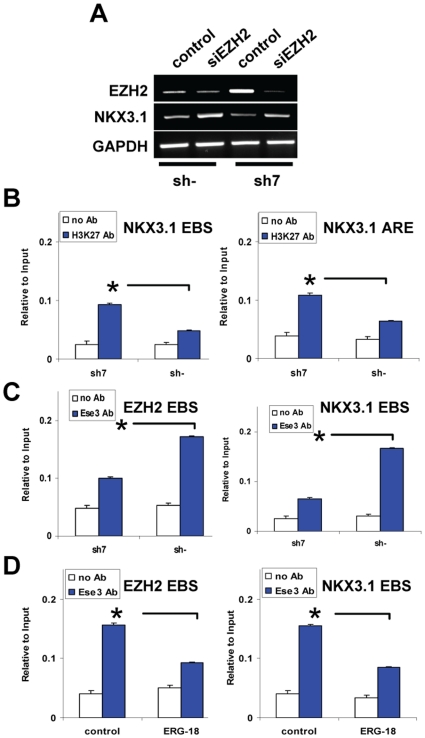
Silencing of Nkx3.1 is mediated by EZH2 in ESE3 knock-down cells. **A.** RNA was extracted from control (sh-) and ESE3 knock-down (sh7) LNCaP cells 48 h post-transfection with siEZH2 and control siRNA and analyzed by RT-PCR. **B.** H3K27 methylation was assessed in sh- and sh7 LNCaP cells by ChIP and qPCR with primers encompassing the EBS and ARE in the Nkx3.1 gene. ETS2 was used as negative control (Fig. S8B) **C.** ESE3 binding to the EZH2 and Nkx3.1 promoter was assessed by ChIP and qPCR in sh- and sh7 LNCaP cells. ETS2 was used as negative control (Fig. S9A). **D.** ESE3 binding to the EZH2 and Nkx3.1 promoter in control and ERG-expressing (ERG-18) LNCaP cells assessed by ChIP and qPCR. ETS2 was used as negative control (Fig. S9B). *, p<0.01.

To determine whether the induction of EZH2 consequent to the loss of ESE3 would lead also to Nkx3.1 repression in ESE3-*kd* LNCaP cells, we knocked-down EZH2 in these cells. Down-regulation of EZH2 in ESE3-*kd* LNCaP cells restored the expression of Nkx3.1 to a level similar to parental cells (Fig. 6B). Furthermore, the Nkx3.1 promoter acquired H3K27 methylation in *ESE3-kd* cells (Fig. 6C), confirming that silencing of the gene was also mediated by EZH2. To determine whether CpG promoter methylation was involved in the silencing of Nkx3.1, we analyzed the CpG methylation state in the Nkx3.1 promoter in ESE3-*kd* LNCaP cells. Bisulfite sequencing showed the absence of CpG methylation in the Nkx3.1 promoter in both parental and ESE3-*kd* LNCaP cells (Fig. 4I) ruling out DNA methylation as a contributing factor.

The changes observed in EZH2 and Nkx3.1 expression in *ESE3-kd* cells suggested that ESE3 could bind to the promoter of these genes and act alternatively as a transcriptional activator or repressor. To test this hypothesis, we performed ChIP to assess binding of ESE3 to the regions of the EZH2 and Nkx3.1 promoters containing the identified EBS in parental and ESE3-*kd* LNCaP cells. As shown in Fig. 6C (*right panel*), binding of ESE3 to the Nkx3.1 promoter was seen in parental and not in ESE3-*kd* LNCaP cells, supporting the idea that ESE3 acted as a transcriptional activator of this gene. ChIP showed also that ESE3 was bound to the EZH2 promoter in parental LNCaP cells, while binding was decreased in ESE-*kd* cells. In this case, binding of ESE3 would have a negative effect on EZH2 transcription (Fig. 6C, *left panel*).

These data indicated that ERG and ESE3 could bind to overlapping sites in the EZH2 and Nkx3.1 promoters and regulate their transcription in opposite directions. Thus, ERG and ESE3 might compete with each other for promoter occupancy, switching alternatively on and off transcription. To test this hypothesis, we performed ChIP to assess binding of ESE3 to the promoters in parental and ERG-expressing LNCaP cells. ESE3 was bound to the EZH2 promoter in parental cells, but its presence was significantly reduced in ERG expressing cells, indicating that ERG could displace ESE3 from the EZH2 promoter (Fig. 6D, *left panel*). Consistently, ChIP showed also that binding of ESE3 to the Nkx3.1 promoter was reduced in ERG over-expressing compared to parental LNCaP cells (Fig. 6D, *right panel*). Thus, ERG and ESE3 competed with each other for binding to these promoters. ESE3 mediated activation of Nkx3.1 and repression of EZH2 could be reversed by ERG when over-expressed in prostate cells by direct competition for promoter occupancy. Altogether, these data support the existence of an ETS transcriptional network that controls expression of key target genes involved in cell proliferation and differentiation.

### Aberrant Expression of ERG and ESE3 Increases Cell Migration and Anoikis

**Figure 7:**
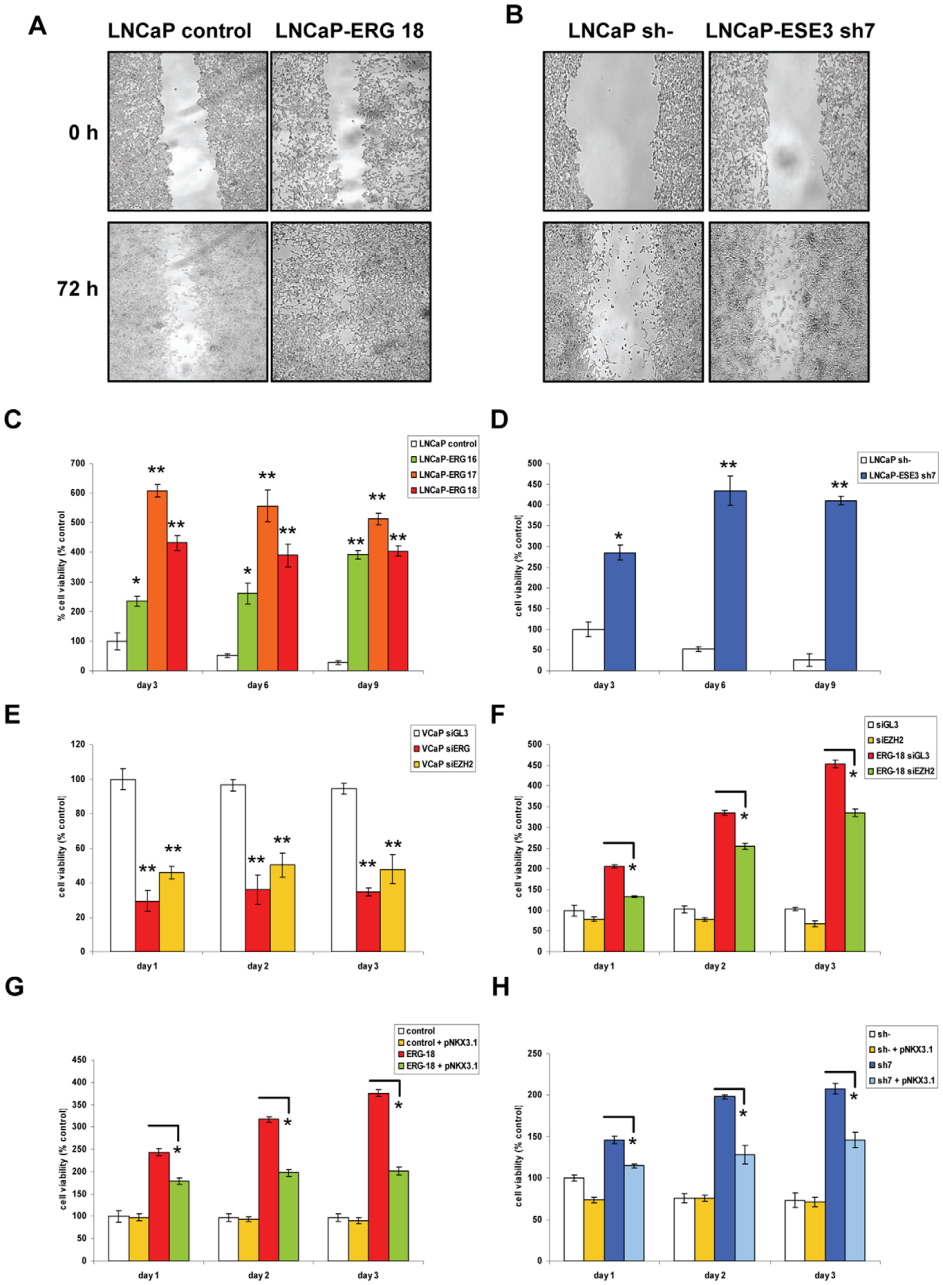
Effects of deregulated expression of ERG and ESE3 in prostate cancer cells. **A.** ERG over-expression and ESE3 knock-down **(B)** enhance cell migration. Cells were grown until confluence and starved for 24 h when a scratch was performed on the monolayer. Pictures were taken at 0 and 72 h. Representative photographs of triplicate experiments are shown. **C.** Control and ERG over-expressing LNCaP cell clones were plated in polyhema coated 96-well plates and cell viability was measured using a colorimetric assay at the indicated time points. **D.** Control and ESE3 knock-down LNCaP cells were plated in polyhema and assayed as described above. **E.** VCaP cells were transfected with siRNAs against ERG or EZH2 and plated in polyhema after 24 h. Cell viability was measured as described above. **F.** Parental and ERG-expressing (ERG-18) LNCaP cells were transfected with siRNAs against EZH2 and assayed as indicated above. **G.** Parental and ERG-expressing (ERG-18) LNCaP cells were transfected with full length Nkx3.1 expression vector and assayed as indicated above. **H.** Parental (sh-) and ESE3 knock-down (sh-7) LNCaP cells were transfected with full length Nkx3.1 expression vector and assayed as indicated above. Data are presented as mean ± SD of triplicate experiments. *, p<0.01; **, p<0.001.

Functional annotation analysis of the transcriptome of ERG^high^ and ESE3^low^ tumors linked both ERG and ESE3 to critical processes in tumor initiation and progression and suggested that their deregulation might induce partially overlapping features. To understand the functional consequences of altered expression of ERG and ESE3, we evaluated the effects of ERG over-expression and ESE3 knock-down on cell migration using the *in vitro* scratch/wound healing assay and on cell survival in non-adherent conditions (anoikis assay). Resistance to anoikis, along with increased cell motility, contributes to cancer cell dissemination and metastasis. ERG expression increased cell migration of LNCaP cells that have limited motility (Fig. 7A). LNCaP cells with ESE3 knock-down exhibited also increased cell migration compared to parental LNCaP cells (Fig. 7B). Resistance to anoikis was increased both in ERG over-expressing and ESE3 knock-down LNCaP cells (Fig. 7C-D). These findings support the hypothesis that dysregulated expression of ERG and ESE3 induced similar phenotypes in prostate epithelial cells.

Transient knock-down of ERG in VCaP cells decreased survival in anoikis conditions (Fig. 7E). To assess the contribution of EZH2 induction to this cell phenotype, we transiently knock-down EZH2 in VCaP (Fig. 7E) and parental and ERG-expressing LNCaP cells (Fig. 7F). EZH2 knock-down significantly reduced resistance to anoikis in both types of ERG expressing cells while it did not affect parental LNCaP cells (Fig. 7E-F), indicating that EZH2 mediated at least in part the effects of aberrantly expressed ERG. To assess also the contribution of Nkx3.1 to this malignant phenotype, we transiently expressed Nkx3.1 in parental and ERG-expressing LNCaP cells (Fig. 7G). Transient expression of Nkx3.1 significantly reduced survival in anoikis condition of ERG-expressing LNCaP cells without any effect in parental LNCaP cells, which endogenously express Nkx3.1. A similar result was obtained by transient expression of Nkx3.1 in ESE3 knock-down LNCaP cells with a significant reduction of their ability to survive in anoikis condition (Fig. 7H). Collectively, these data indicated that both EZH2 and NKx3.1 mediated relevant effects of ERG and ESE3 in prostate cancer cells.

## Discussion

ETS transcription factors regulate important signaling pathways involved in cell differentiation and development in many tissues and have emerged as important players in pathogenesis of epithelial and non-epithelial tumors [10]. ETS factors have recently attracted significant attention in prostate cancer since the identification of recurrent ETS gene rearrangements leading to their over-expression [2], [3], [4]. This study provides new insights into the relevance of the ETS transcriptional network in the prostate and identifies a link between ETS factors, epigenetic pathways and expression of tissue-specific differentiation and tumor suppressor genes.

We found that dysregulated expression of ETS factors with putative oncogenic and tumor suppressor properties was very frequent, with up to 80% of prostate tumors having one or more aberrantly expressed ETS gene. Thus, our study indicates that prostate tumors truly without altered ETS gene expression (NoETS) represent a relatively minor group. This is a relevant finding made possible by the QRT-PCR assessment of the expression level of multiple ETS genes in cancer and normal prostate tissue samples, which to our knowledge had not been evaluated before. Most tumors, which would have been classified as ETS negative based on the exclusive assessment of the few ETS genes known to be translocated in prostate tumors (i.e., ERG, ETV1 and ETV4) had in fact significantly alterations of other ETS factors. ESE3 and ESE1 were the most frequently affected ETS genes. ESE3 and ESE1 are normally expressed in prostate epithelial cells and their expression was significantly altered (≥4-fold relative to normal prostate) in >40% of cases, including many tumors with ERG translocation and over-expression. ESE3 and ESE1 have been shown to act as a tumor suppressor [11] and oncogene [12], respectively, and thus can have a relevant impact on prostate tumorigenesis. The mechanism of altered expression of these ETS factors in prostate tumors is unknown at this time. Epigenetic events and environmental stress (e.g., inflammation) might be involved, since we showed that ESE3 is epigenetically silenced in prostate cancer cells [11] and both ESE3 and ESE1 expression can be affected by inflammatory stimuli [33].

To decipher the network of genes controlled by the aberrantly expressed ETS factors we divided prostate tumors in groups on the basis of their ETS expression profile. This approach has been applied in previous studies [27], [34]. The major difference in our work is that we took in consideration all the ETS genes that had shown relevant changes in expression and divided tumors according to their ETS expression status evaluated by real time PCR instead of relying only on microarray data. Using stringent criteria we defined four major prostate tumors subgroups. Principal components and functional annotation analyses indicated that the tumor subgroups exhibited distinct transcriptional and biological features. Among the subgroups, ERG^high^ and ESE3^low^ tumors had the most robust transcriptional signatures with many distinctive features compared to normal prostate and NoETS tumors. Functional annotation analysis pointed to a strong impact of these dysregulated ETS factors on the prostate cancer transcriptome with specific enrichment of genes linked to cell adhesion, invasion and migration, which might confer a more aggressive phenotype to these tumors. ETS factors have been reported to play an important role in extra-cellular matrix remodeling and epithelial-to-mesenchymal transition and their over-expression has been linked to increased motility, invasion and metastasis in various cancer models [35], [36]. Notably, the functional annotation analysis indicated that similar consequences on the cancer transcriptome derived from the over-expression of ERG and loss of ESE3 and that these two ETS factors probably could act in part through common molecular pathways. Functional assays supported this hypothesis as ERG gain and ESE3 loss affected similarly cell properties like cell migration and survival in anchorage-independent conditions.

One of the aims of the study was to identify key genes that could mediate effects of the dysregulated ETS factors. We focused on EZH2 that was exclusively up-regulated in ERG^high^ and ESE3^low^ tumors compared to normal prostate and significantly correlated and anticorrelated with ERG and ESE3, respectively. EZH2 is a key element of the Polycomb Repressive Complex 2 (PRC2) and is responsible for the establishment of the repressive H3K27 methylation mark [17], [18]. EZH2 is up-regulated in many cancers, including clinically localized and metastatic prostate cancers [19], [37]. However, the molecular mechanisms by which EZH2 contributes to prostate cancer initiation and progression as well as the factors controlling its expression in this context remain largely unknown. Recent studies have shown that EZH2 is a transcriptional target of pRB-E2F, while microRNA miR-26a and miR-101 control it post-transcriptionally [20], [21], [22], [23]. Here, we show that ERG and ESE3 reciprocally control EZH2. To support our findings, we performed experiments in prostate cell lines in which we experimentally modulated ERG expression. Both up- and down-regulation of ERG affected the expression of EZH2 in the predicted way. Furthermore, ChIP showed binding of ERG to the EZH2 promoter, suggesting that ERG could direct transcription of EZH2. EZH2 promoter occupancy by ERG was also demonstrated in prostate cancer clinical samples, providing in vivo evidence of this interaction. Interestingly, we found that ESE3 controlled EZH2 level in the opposite direction. We observed that there was an inverse relation between ESE3 and EZH2 expression in prostate tumors. Experiments carried out in stable ESE3knock-down cells confirmed that ESE3 negatively regulated EZH2. Intriguingly, ChIP showed that ESE3 was able to bind to the EZH2 promoter, suggesting that it could act as a transcriptional repressor of the gene. Furthermore, binding of ESE3 was reduced in ERG expressing cells, indicating that a direct competition for EZH2 promoter occupancy might explain the reciprocal regulation of EZH2 by these two ETS factors.

EZH2 is a key factor in the execution of development and differentiation programs as well as in maintenance of pluripotency and self-renewal of stem cells [38], [39]. Thus, induction of EZH2 by ETS factors could have important biological consequences and contribute to altered developmental programs and neoplastic transformation of prostate epithelial cells. EZH2 has been shown to control also genes involved cell adhesion, invasion and migration [40], [41], pathways that we found highly enriched in ERG^high^ and ESE3^low^ tumors by functional annotation analysis. The transcriptomes of both ERG^high^ and ESE3^low^ tumors were characterized by a large number of down-regulated genes. This “repressive” gene signature could be attributed to the dysregulated activation of epigenetic effectors like EZH2 by ETS factors leading to silencing of genes with potential anti-tumor activity [37]. We show that dysregulated expression of ERG and ESE3 was associated with reduced expression of the Nkx3.1 tumor suppressor gene. Nkx3.1 is a prostate-specific homeobox protein involved in prostate development and differentiation and is one of the earliest markers of prostate epithelial cell differentiation [25]. Nkx3.1 acts as a prostate-specific tumor suppressor and its loss has an important role in tumor initiation and progression to invasive disease [25]. This transcription factor integrates multiple signaling pathways including PTEN/PI3K/AKT, p53 and AR, which all play critical roles in prostate development and tumorigenesis [25], [42]. Thus, the concomitant induction of EZH2 and attenuation of Nkx3.1 can explain the activation of a broad dedifferentiation program observed in the transcriptome of ERG^high^ and ESE3^low^ tumors. Re-expression of Nkx3.1 in PC3 cells, in which the gene is silenced, inhibited cell proliferation and invasion [43]. In contrast, loss of Nkx3.1 has been associated with transformation and activation oncogenic pathways [44]. We have observed that re-expression of Nkx3.1 in ERG and ESE3 cell models significantly reduces survival in anoikis indicating that Nkx3.1 attenuation mediates at least in part the transforming effects of ERG gain and ESE3 loss. Several mechanisms have been shown to contribute to the loss of Nkx3.1 in prostate cancers including allelic loss, methylation and post-transcriptional control [26], [28]. Here we described an additional pathway leading to its silencing. We show that Nkx3.1 is directly controlled by ERG and ESE3 in prostate tumors and EZH2 contributes to its silencing. ERG bound to the Nkx3.1 promoter and binding was associated with transcriptional repression as shown also by promoter reporter assay. Moreover, the Nkx3.1 promoter acquired the repressive histone H3K27 methylation mark in an ERG-dependent manner and expression of Nkx3.1 increased upon EZH2 knock-down, indicating that EZH2 had an important role in Nkx3.1 silencing in stably ERG expressing cells. On the other hand, we show that Nkx3.1 expression was activated by ESE3 and that stable knock-down of ESE3 significantly reduced the expression of Nkx3.1. Thus, ESE3 influenced the expression of Nkx3.1 by binding to the gene promoter and acting as a transcriptional activator. At the same time, ESE3 could prevent Nkx3.1 silencing by repressing EZH2 and blocking H3K27 methylation. Interestingly, silencing of Nkx3.1 upon ERG over-expression and ESE3 down-regulation occurred independently of CpG promoter methylation, consistent with a major role of EZH2 and H3K27 methylation. Moreover, while Nkx3.1 expression was controlled positively by ESE3, ERG abolished this effect by competing with ESE3 for Nkx3.1 promoter occupancy and increasing EZH2 expression. Thus, these findings provide a mechanistic explanation for the attenuation of Nkx3.1 expression in ERG^high^ and ESE3^low^ tumors and suggest that this might be a general mechanism to repress tumor suppressor genes by aberrantly expressed ETS factors. To date, Polycomb responsive elements have not been defined in mammalian gene cells. It has been suggested that transcription factors may contribute to the recruitments of EZH2 on target genes and that this phenomenon is context dependent [31]. Consistently with this hypothesis, our data suggest that deregulated expression of ERG and ESE3 may affect EZH2 recruitment and H3K27 methylation of target gene promoters. Future studies will be necessary to determine whether and how ETS factors might direct EZH2 to selected gene promoters.

**Figure 8:**
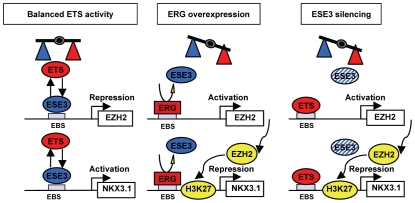
Model of the reciprocal regulation of EZH2 and Nkx3.1 by competing ETS factors. ESE3 controls expression of EZH2 and Nkx3.1 in normal prostate epithelial cells. ERG over-expression or loss of ESE3 leads to abnormal expression of these key genes and promotes cell transformation.

Collectively, this work provides novel insights into the complex role of ETS factors in prostate development and tumorigenesis. Our findings support the model of an ETS transcriptional network whose balance regulates the expression of key genes involved in prostate epithelial cell development and differentiation and whose disruption can lead to tumorigenesis (Fig. 8). We show that the epithelial-specific ETS factor ESE3 promotes the expression of tissue-specific differentiation genes like Nkx3.1 in prostate epithelial cells, while it represses genes with transforming potential like EZH2. Furthermore, an oncogenic ETS factor, like ERG, binds to the promoter of EZH2 and Nkx3.1 and competes with ESE3 for promoter occupancy reversing its effects. Thus, ESE3 could be a critical factor to maintain the equilibrium between competing stimuli and allow the correct developmental and differentiation programs to proceed. Genetic events or pathological conditions, such as gene rearrangements [3] or chronic inflammation [1], could shift the equilibrium in favor of oncogenic ETS, like ERG and ESE1, and promote the activation of pro-mitogenic, pro-survival and dedifferentiation programs. In this context, it is possible that altered expression of ETS factors, like ESE3 and ESE1, which are normally present in prostate epithelial cells, might represent an early event, cooperating with or even preceding ETS gene rearrangements in the early stages of prostate tumorigenesis.

## Materials and Methods

### Clinical Sample Collection

Tissue samples and clinical data were collected with the approval of the Ethics Committee of the Piedmont Region, Italy, and patients' written informed consent. Tumor samples (*n = 65*) were taken from patients undergone to radical prostatectomy. Normal prostate tissue (*n = 14*) was taken from adult male who underwent multiple diagnostic prostate biopsies and found to be disease-free [11]. Age ranges of individuals with cancer and normal prostate were 50–74 and 48–78 years, respectively. Clinical parameters such as Gleason, tumor stage, and PSA values were recorded at the time of surgery. Association between clinical variables and tumor classification according to ETS expression status was tested with Fisher test available within R statistical package.

### Microarray Analysis

RNA extracted from tissue samples was amplified and labeled using Ambion Message Amp I and hybridized on Agilent Human 1A glass arrays using a dye-swap replication scheme as described [11]. Arrays were scanned with the Agilent B scanner and raw data files were loaded into the Resolver SE System (Rosetta Biosoftware) for data normalization and processing applying the Agilent platform-specific error model. A commercial pool of RNA from organ donor healthy prostates (Becton Dickinson) was used as common reference. A log2 gene expression matrix was created after combining dye-swap replicates. Expression data were filtered for SD>0.5 across the samples, resulting in 5142 probes. Microarray data are MIAME compliant and have been deposited in the Gene Expression Omnibus (GEO accession no. GSE14206).

### Quantitative Real-Time PCR

RNA was reverse-transcribed and real-time PCR was performed using custom made primers and SYBR Green chemistry for ESE3 [11] and commercial primer sets for ERG, ESE1, ETS2, ETS1, ESE2 and PDEF (Applied Biosystems) on a ABI 7000 system. Samples were analyzed in triplicate. The level of each gene was calculated by comparing the *Ct* value in the samples to a standard curve generated from serially diluted cDNA from a reference sample and normalizing it to the amount of β-actin as previously described [11].

### Identification of Tumor Subgroups

Tumors were grouped according to the predominantly expressed ETS factor based on QRT-PCR data and considering a cut-off of ≥4 fold change compared to the average value in normal prostates. Applying this cut-off, up to 80% of tumors had highly deregulated expression of at least one ETS gene and were divided into four major subgroups: ERG^high^ (*n = 14*), ESE1^high^ (*n = 12*), ESE3^low^ (*n = 13*) and NoETS (*n = 14*) tumors. Eight tumors with high level either of ETV1, ETV4, ETS2 or ETS1 were excluded from the analysis because they did not fit in any of the other categories and were too heterogeneous to be analyzed separately. Microarray data from an independent study [14] were downloaded from the GEO public repository (GSE6956). This set contained 69 prostate cancers profiled using the Affymentrix GeneChip HG-U133A 2.0 arrays. Raw Affymetrix CELL files were processed applying the robust multi-array average (RMA) procedure within the Affy R package. Expression log intensities of ERG, ESE3, ESE1, ESE2, ETS2, ETS1 and PDEF were recorded and samples were classified as ERG^high^, ESE3^low^, and ESE1^high^, when the level of these genes was at least 3 fold higher or lower than level observed in NoETS samples.

### Principal component analysis

Principal component analysis was performed on the matrix the 5142 filtered genes with highest standard deviation using MeV version 4.2 (http://www.tm4.org/mev.html). Points representing individual tumors and normal samples were plotted in a 2D scatter plot where the coordinates correspond to the first two principal components (combinations of genes).

### Differential Gene Expression and Functional Annotation Analysis

Differential gene expression analysis between sample classes was performed on the matrix containing the 5142 filtered genes using Gene Expression Profile Analysis Suite (GEPAS). Differentially expressed genes were obtained after filtering for q value (Q ≤0.1). To test the difference in gene set overlap, we crossed the lists of up-regulated and down-regulated genes of each class using a standard Venn diagram generator. To test the statistical significance of the differences in shared and distinctive features, we built for each class pair contingency tables containing observed and expected overlap and applied the Fisher Exact test. To define the pathways affected in tumor subgroups the lists of differentially expressed genes obtained with GEPAS for each tumor subgroup compared to normal prostate were uploaded into the GeneGO's Metacore server (http://portal.genego.com). The Metacore software for integrated functional analysis of gene expression data compare distinct datasets to determine biological features shared or unique to each set. The matrix combining the results from the four class comparisons (genes with q value ≤0.1 in at least one class) was analyzed using the Metacore “Compare Experiments Workflow” tool to visualize enriched features in GeneGo pathway maps (GGPM). To identify genes correlated and anti-correlated with ERG and ESE3 we used the R function cor.test( ), which provides Pearson's correlation measure along with a P value estimate.

### Cell Cultures, Cell Transfection and Selection of Stable Cell Clones

VCaP, LNCaP, 22Rv1 and PC3 were obtained from American Type Culture Collection (ATCC, Manassas, VA, USA) and maintained in DMEM (VCaP) or RPMI-1640 (all others) supplemented with 10% fetal bovine serum. VCaP cells are ERG translocation positive, AR-positive and androgen dependent [2]. LNCaP cells are AR-positive, androgen-dependent and carry the ETV1 gene translocation [4]. 22Rv1 cells are ETS-translocation negative, AR-positive and androgen-independent [45]. Immortalized prostate epithelial LHS cells, which have been engineered to express hTERT and SV40 large T antigen [32], were maintained in PrEC growth medium (PrEGM, Cambrex, Lonza Group Ltd, Basel, Switzerland). ERG expressing stable cell lines were generated by transfection of the pECFL-ha-ERG3 expressing vector (provided by S. Izraeli) using Lipofectamine 2000 (Invitrogen) and selection with G418. Negative control cells were obtained by transfection with pcDNA3.1 and selection in G418. To establish stable ESE3 knock-down cell lines, cells were transfected with ESE3 targeting shRNAs (Cat. no. KH14264N SuperArray, Frederick, MD, USA) using Lipofectamine 2000 and selected with G418. Negative control cells were generated by transfection of a control shRNA (SuperArray) with no sequence homology within the human genome. In both cases, G418 resistant colonies were expanded and expression of ERG and ESE3 was determined by RT-PCR and Western blotting. Nkx3.1 and EZH2 expression vectors were provided by E. P. Gelmann and J-T. Hsieh, respectively. For transient gene knock-down cells were transfected with siRNAs directed to ERG (Cat. no. Hs_ERG_8, Qiagen, Hilden, Germany), EZH2 (Cat. no. D-004218-01-0005, Dharmacon, Lafayette, CO, USA) or a control siRNA directed to the firefly luciferase gene (siGL3, Ambion [46]). Cells were plated in six-well plates and transfected with 50 nM of siRNA using Interferin (Polyplus-transfection SA, Illkirch, France) and were harvested after 72 h.

### Luciferase Promoter Reporter assay

Cells were plated in 48-well plates and 24 h later transfected with the pGL3-Nkx3.1 promoter reporter (provided by J. M. Bentel) or pGL3-control vector (Promega AG, Wallisellen, Switzerland) along with control empty vector or ESE-3, ERG, EZH2 expression vectors. pRL-SV40 (Promega) was used as control to monitor transfection efficiency. Luciferase activity was measured after 24 h using the Dual-Luciferase Reporter Assay System (Promega) as previously described [11]. Data are presented as percentage of Firefly luciferase activity normalized to the Renilla luciferase activity relative to cells transfected with control vector alone. Reporter assays were performed in triplicate and repeated in three independent experiments.

### Anoikis and Migration Assay

Cell viability in anchorage-independent conditions (anoikis assay) was assessed by plating cells (1×10^5^cells/well) in poly-hema coated 96-well plates. Cell viability was measured using a colorimetric assay (MTT, Sigma) and reading absorbance at 540 nm in a microplate reader. All assays were done in triplicates and in three independent experiments. Cell migration was assessed using the scratch wound healing assay [47]. Cells were grown to confluence in six-well plates and then overnight in serum-free medium. After scratches were performed on the cell monolayer, complete medium was added to the cultures and images were taken at 24–72 h with a Zeiss microscope. The assays were done in triplicate in three independent experiments.

### RT-PCR and Western Blotting

RNA was extracted and end-point RT-PCR was performed using the SuperScript One-step RT-PCR system (Invitrogen) as described previously [11]. PCR primers are shown in Table S4. PCR products were separated by gel electrophoresis and visualized on a AlphaImager 3400 (AlphaInnotech, Fremont, CA, USA). For Western blotting, cell lysate preparation, gel electrophoresis and blotting were performed as described previously [11] using antibodies for ERG (*sc-353*, Santa Cruz Biotechnology Inc., Heidelberg, Germany), ESE-3 (ETS3 Clone 5A5, Lab Vision, Fremont CA. USA), EZH2 (*612667* BD Biosciences, San Jose, USA), Nkx3.1 (*SC-15022,*Santa Cruz Biotechnology Inc.) and α-tubulin (*CP06,* Calbiochem). To detect the TMPRSS2:ERGa fusion transcript, total RNA from tumor (*n = 53*) and normal prostate tissue (*n = 14*) was reverse-transcribed using random hexamers and the cDNA amplified with primers located in exon 1 of TMPRSS2 and exon 4 of ERG (Table S4) and Amplitaq Gold (Applied Biosystems). The TMPRSS2-ERGa specific PCR product was 184 bp long.

### Chromatin Immunoprecipitation (ChIP)

Cells were exposed to formaldehyde to cross-link protein-DNA complexes and processed as previously described [11], [46]. Immuno-precipitation was done with antibodies for ERG (sc-354 X, Santa Cruz Biotechnology Inc., Heidelberg, Germany), ESE-3 (ETS3 Clone 5A5, Lab Vision, Fremont, CA USA)[11], and methylated H3K27 (07-449 Millipore Upstate Biotechnology, NY, USA)[6], [46]. End-point PCR was performed as previously described [11], [46] using primers spanning the EBS in the region of interest (shown in Table S4) and Taq Gold (Roche). PCR products were analyzed by gel electrophoresis. Quantitative real-time PCR (qPCR) was performed using SYBR Green qPCR and the primers indicated in Table S4. The amount of immunoprecipitated DNA was calculated in reference to a standard curve and normalized to input DNA [46]. To perform ChIP in clinical samples, fresh frozen tumor specimens were cut into small pieces, placed in PBS/Na-butyrate/formaldehyde fixative and then immediately processed for ChIP as described above. All experiments in cell lines and tumor specimens were repeated at least three times and representative results are shown.

### Bisulphite Sequencing

Genomic DNA was extracted and bisulphite conversion was performed using the DNeasy Blood and Tissue kit and the Epitect Bisulphite kit (Qiagen AG, Hombrechtikon, CH) as described [11]. Bisulfite-modified DNA was amplified by PCR with primers and sequenced. Primers for bisulphite sequencing PCR are shown in Table S4. The location of the CpG island in the Nkx3.1 locus was determined using the NCBI Map Viewer and the CpG island map option. Primers for bisulphite sequencing PCR were designed using Methprimer to interrogate the greatest number of CpG sites within a single PCR product. PCR products were purified using the JetQuick PCR purification system (Chemie Brunschwig) and sequenced on an ABI 3730xl DNA Analyzer.

## Supporting Information

Table S1 Genes differentially expressed in tumor subgroups.
(0.45 MB XLS)

TableS2 Genes selectively modulated in ERGhigh and ESE3low tumors.
(0.08 MB XLS)

Table S3 Genes correlated and anti-correlated with ERG and ESE3.
(0.17 MB XLS)

Table S4 Primer sets for RT-PCR, ChIP and bisulfite-treated DNA sequencing.
(0.07 MB DOC)

Figure S1 Expression of selected ETS factors evaluated by quantitative real time RT-PCR.
(0.09 MB PDF)

Figure S2 Identification of ETS tumor subgroups in an independent microarray dataset. (A) Expression of ERG, ESE1 and ESE3 in prostate tumors according to microarray data. (B) Patient distribution among the four subgroups. (C) ERG level in NoETS and ERGhigh tumors. (D) ESE3 expression level in NoETS and ESE3low tumors.
(0.13 MB PDF)

Figure S3 TMPRSS2:ERG fusion transcripts in the ERGhigh tumor, normal prostate and benign prostatic hyperplasia samples (A). Patient distribution in the four tumor subgroups according to Gleason score, tumor stage and pre-operatory PSA level (B).
(0.17 MB PDF)

Figure S4 Four-way Venn diagrams showing shared and distinct differentially expressed genes among the four tumor subgroups.
(0.06 MB PDF)

Figure S5 Establishment of cell models for ERG and ESE3 target gene identification. (A) Stable clones of ERG transfected LNCaP and 22Rv1 cells. (B) ERG knock-down in VCaP cells. (C) ERG target genes in ERG expressing 22Rv1 and LNCaP cells. (D) ERG target genes in ERG-knock-down VCaP cells. (E) Stable ESE3 knock-down LNCaP and LHS cells.
(0.23 MB PDF)

Figure S6 Positive control experiments for ChIP assays in VCaP, parental and ERG expressing LNCaP and 22Rv1 cells.
(0.07 MB PDF)

Figure S7 Negative control experiments for ChIP assays in ERG expressing and non-expressing cell lines and in ERGhigh and NoETS tumors.
(0.04 MB PDF)

Figure S8 Negative control experiments for ChIP assays in parental and ERG-expressing LNCaP cells and parental and ESE-kd LNCaP cells.
(0.04 MB PDF)

Figure S9 Negative control experiments for ChIP assays in parental and ESE-kd LNCaP cells and parental and ERG-expressing LNCaP cells.
(0.04 MB PDF)
