# Supplementary material for: ETS Transcription Factors Control Transcription of EZH2 and Epigenetic Silencing of the Tumor Suppressor Gene Nkx3.1 in Prostate Cancer
Source: PLoS One. 2010 May 10;5(5):e10547. doi: 10.1371/journal.pone.0010547 (PMC2866657; doi:10.1371/journal.pone.0010547)
Supplement: Table S4 — Primer sets for RT-PCR, ChIP and bisulfite-treated DNA sequencing. (0.07 MB DOC) [file pone.0010547.s004.doc]

**Supplementary Table S4.**

**Primer sets for RT-PCR, ChIP and bisulfite-treated DNA sequencing.**

| Assay | Gene/Region | Sequence 5’ to 3’ | Bases | Primer |
| --- | --- | --- | --- | --- |
| RT-PCR | *ERG* | gacgacttccagaggctcac | 613 - 632 | ERG_RT_f |
| RT-PCR | *ERG* | gcactgtggaaggagatggt | 915 - 934 | ERG_RT_r |
| RT-PCR | *GAPDH* | ggctgggcaaggtcatcc | 644 - 633 | GAPDH_RT_f |
| RT-PCR | *GAPDH* | tccaccaccctgttgctgta | 955 - 972 | GAPDH_RT_r |
| RT-PCR | *CRISP3* | gctgcttccatcttttccag | 54 - 73 | CRISP3_RT_f |
| RT-PCR | *CRISP3* | atttgctgcagcctctttgt | 212 - 231 | CRISP3_RT_r |
| RT-PCR | *MMP3* | gcagtttgctcagcctatcc | 102 - 121 | MMP3_RT_f |
| RT-PCR | *MMP3* | gagtgtcggagtccagcttc | 296 - 315 | MMP3_RT_r |
| RT-PCR | *PLA1A* | cacagacaccgacaatttgg | 682 - 701 | PLA1A_RT_f |
| RT-PCR | *PLA1A* | caaggaaggccttgtagctg | 880 - 899 | PLA1A_RT_r |
| RT-PCR | *EZH2* | aggacggctcctctaaccat | 1710 - 1729 | EZH2_RT_f |
| RT-PCR | *EZH2* | cttggtgttgcactgtgctt | 1869 - 1888 | EZH2_RT_r |
| RT-PCR | *NKX3.1* | gtacctgtcggcccctgaacg | 489 - 509 | NKX3.1_RT_f |
| RT-PCR | *NKX3.1* | gctgttatacacggagaccagg | 675 - 696 | NKX3.1_RT_r |
| RT-PCR | *ESE3* | tgcagcatctgaagtggaac | 434 - 453 | ESE3_RT_f |
| RT-PCR | *ESE3* | aggaaggtgactggtggttg | 633 - 652 | ESE3_RT_r |
| RT-PCR | *TMPRSS2* | taggcgcgagctaagcag | 1367 - 1386 | TMPRSS2_EX1_f |
| RT-PCR | *ERG* | gtccatagtcgctggaggag | 1367 - 1386 | ERG_EX4_r |
| CHIP | *EZH2* | agaccagcctgaccaagacc | -862 - -843 | EZH2_EBS1_f |
| CHIP | *EZH2* | gagtttcgctctggttgtcc | -687 - -678 | EZH2_EBS1_r |
| CHIP | *MMP3* | cctctaccaagacaggaagca | -227 - -207 | MMP3_EBS_f |
| CHIP | *MMP3* | aggaaggtgactggtggttg | -112 - -93 | MMP3_EBS_r |
| CHIP | *NKX3.1* | ggaaccaccaaagaaaacca | -607 - -588 | NKX3.1_EBS_f |
| CHIP | *NKX3.1* | gagccaattacaggggatga | -402 - -383 | NKX3.1_EBS_r |
| CHIP | *NKX3.1* | ttgcataaattaggggagaacatacca | 5733 - 5707 | NKX3.1_ARE_f |
| CHIP | *NKX3.1* | gagggacccagctgcgattca | 5306 - 5286 | NKX3.1_ARE_r |
| CHIP | *ETS2* | tctctcctccctcgtttcct | -106 - -87 | ETS2_f |
| CHIP | *ETS2* | tcgtcagtctctggaggaagt | 8 - 28 | ETS2_r |
| Sequ. | *NKX3.1* | atttaggtgggtaagagagggttt | 145 - 164 | NKX3.1_BS_f |
| Sequ. | *NKX3.1* | caataacaaaacaaaaattaaccaaca | 145 - 164 | NKX3.1_BS_r |
| Real Time- CHIP | *EZH2* | ctgaggcatgagaatcgcttga | -773 - -752 | EZH2_EBS_f |
| Real Time- CHIP | *EZH2* | agacggagtttcgctctggttg | -694 - -673 | EZH2_EBS_r |
| Real Time- CHIP | *NKX3.1* | tgcggataaaggaaccacca | -617 - -601 | NKX3.1_EBS_f |
| Real Time- CHIP | *NKX3.1* | aggcatgacaagtaggtgcagc | -528 - -507 | NKX3.1_EBS_r |
| Real Time- CHIP | *NKX3.1* | tgaaaagcatgccctggtg | 5541 - 5559 | NKX3.1_ARE_f |
| Real Time- CHIP | *NKX3.1* | cgcggtgagaaaatcagtgtc | 5438 - 5446 | NKX3.1_ARE_r |
| Real Time- CHIP | *MMP3* | cctctaccaagacaggaagca | -227 - -207 | MMP3_EBS_f |
| Real Time- CHIP | *MMP3* | aggaaggtgactggtggttg | -112 - -93 | MMP3_EBS_r |
| Real Time- CHIP | *ETS2* | tctctcctccctcgtttcct | -106 - -87 | ETS2_f |
| Real Time- CHIP | *ETS2* | tcgtcagtctctggaggaagt | 8 - 28 | ETS2_r |
