# Supplementary figures and images for: ETS Transcription Factors Control Transcription of EZH2 and Epigenetic Silencing of the Tumor Suppressor Gene Nkx3.1 in Prostate Cancer
Source: PLoS One. 2010 May 10;5(5):e10547. doi: 10.1371/journal.pone.0010547 (PMC2866657; doi:10.1371/journal.pone.0010547)

Kunderfranco et al Supplemental Figure S1

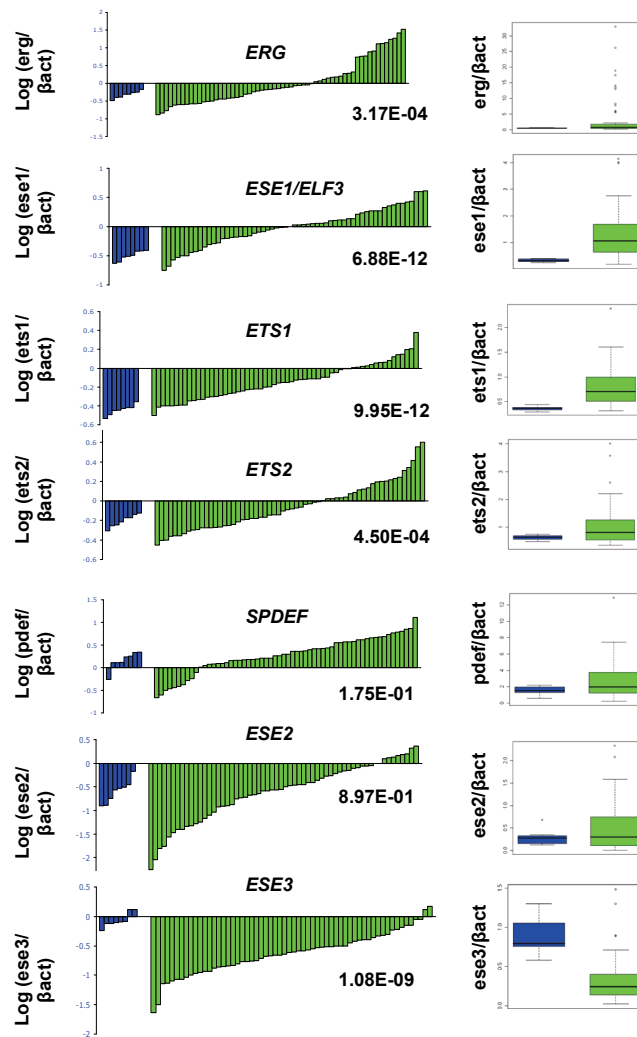

Supplement: Figure S1 — Expression of selected ETS factors evaluated by quantitative real time RT-PCR. (0.09 MB PDF) [file pone.0010547.s005.pdf]

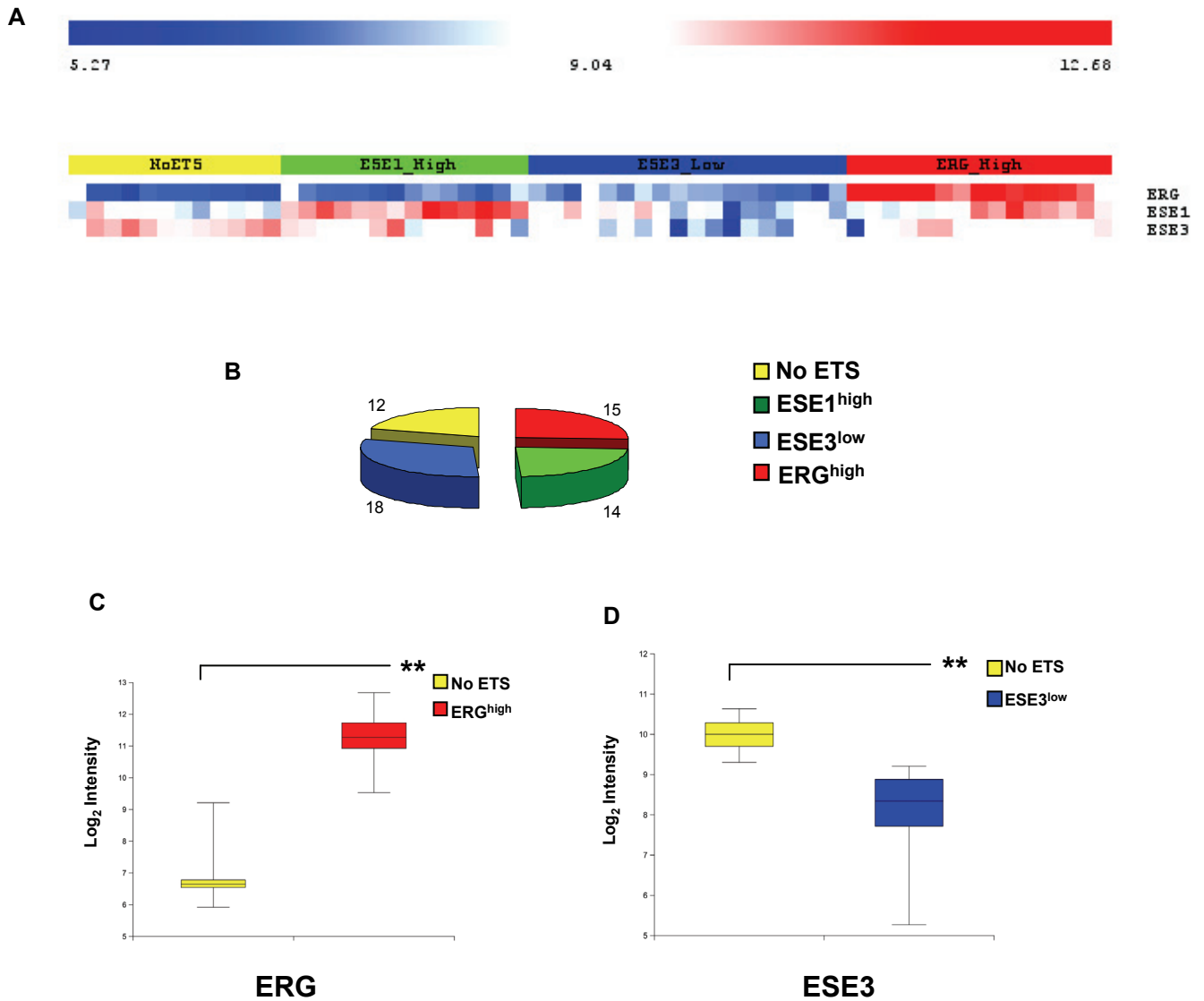

Supplement: Figure S2 — Identification of ETS tumor subgroups in an independent microarray dataset. (A) Expression of ERG, ESE1 and ESE3 in prostate tumors according to microarray data. (B) Patient distribution among the four subgroups. (C) ERG level in NoETS and ERGhigh tumors. (D) ESE3 expression level in NoETS and ESE3low tumors. (0.13 MB PDF) [file pone.0010547.s006.pdf]

B

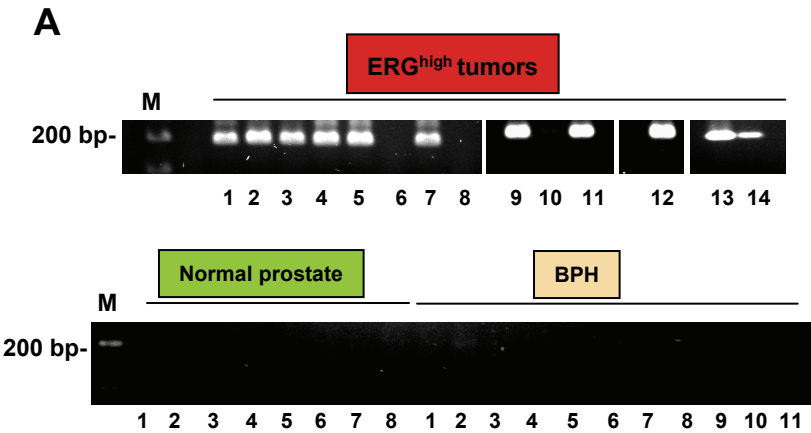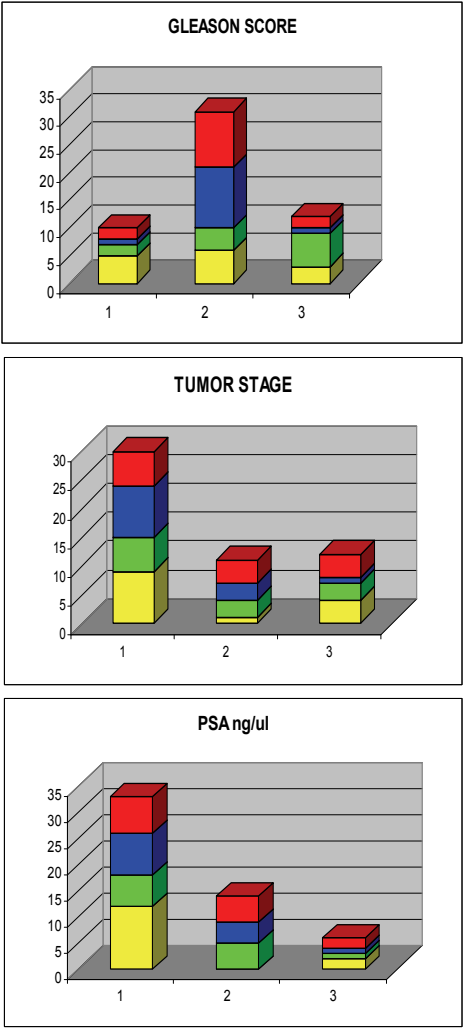

Histopathological parameters

Gleason Score

|   |    |
|---|----|
| 1 | ≤6 |
| 2 | 7  |
| 3 | >7 |

Tumor Stage

|   |        |
|---|--------|
| 1 | ≤T2C   |
| 2 | T3A-B  |
| 3 | T3C-T4 |

PSA (ng/ul)

|   |        |
|---|--------|
| 1 | ≤10    |
| 2 | >10-20 |
| 3 | >20    |

Supplement: Figure S3 — TMPRSS2:ERG fusion transcripts in the ERGhigh tumor, normal prostate and benign prostatic hyperplasia samples (A). Patient distribution in the four tumor subgroups according to Gleason score, tumor stage and pre-operatory PSA level (B). (0.17 MB PDF) [file pone.0010547.s007.pdf]

GENES UPREGULATED

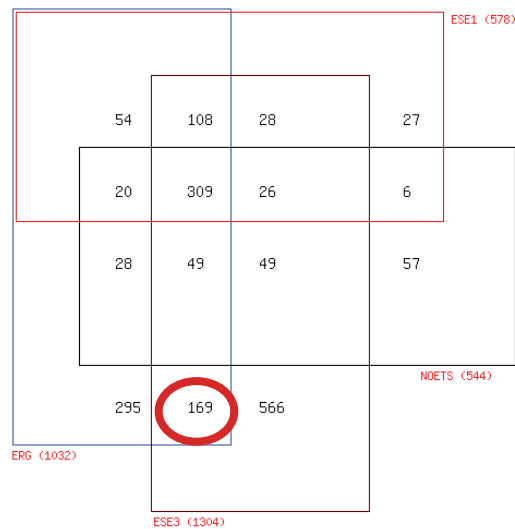

GENES DOWNREGULATED

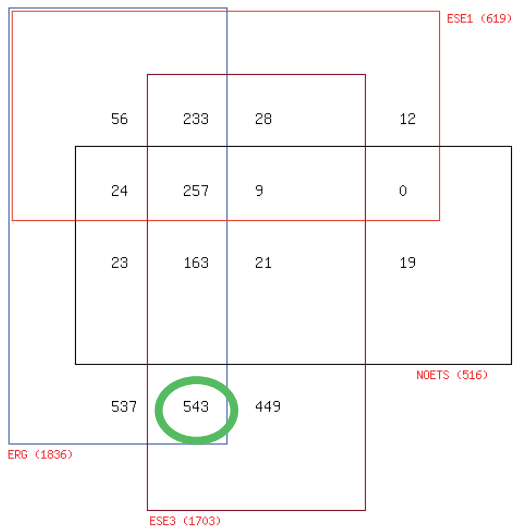

Supplement: Figure S4 — Four-way Venn diagrams showing shared and distinct differentially expressed genes among the four tumor subgroups. (0.06 MB PDF) [file pone.0010547.s008.pdf]

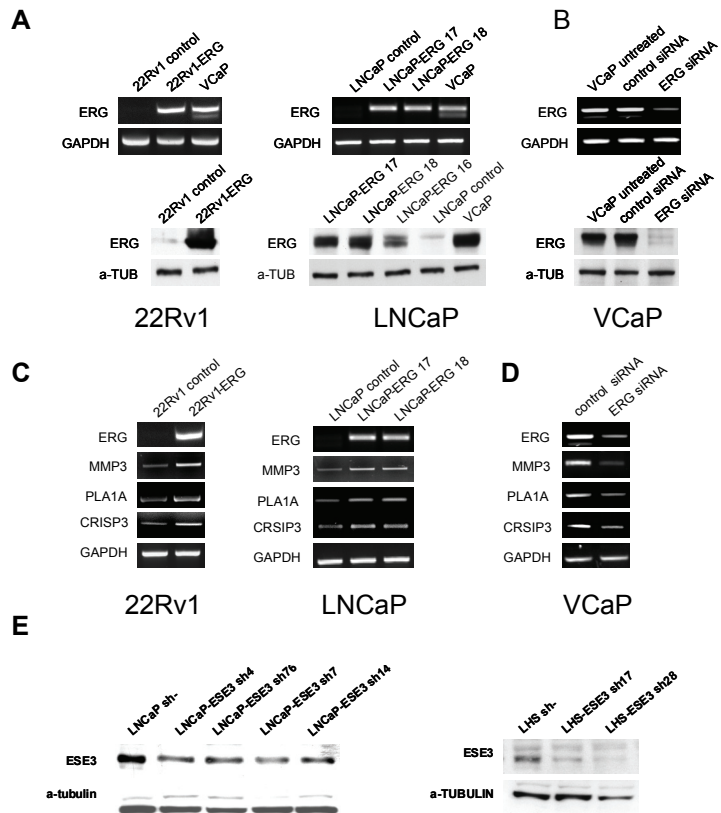

Supplement: Figure S5 — Establishment of cell models for ERG and ESE3 target gene identification. (A) Stable clones of ERG transfected LNCaP and 22Rv1 cells. (B) ERG knock-down in VCaP cells. (C) ERG target genes in ERG expressing 22Rv1 and LNCaP cells. (D) ERG target genes in ERG-knock-down VCaP cells. (E) Stable ESE3 knock-down LNCaP and LHS cells. (0.23 MB PDF) [file pone.0010547.s009.pdf]

**A**

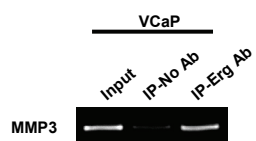

**B**

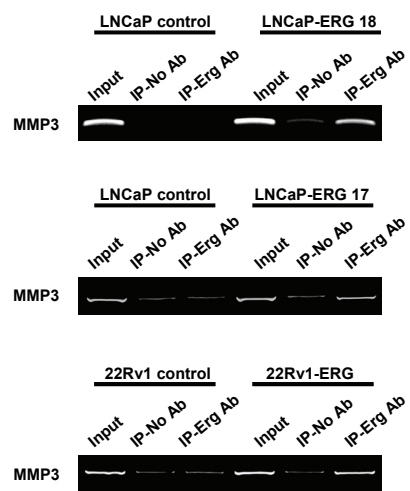

Supplement: Figure S6 — Positive control experiments for ChIP assays in VCaP, parental and ERG expressing LNCaP and 22Rv1 cells. (0.07 MB PDF) [file pone.0010547.s010.pdf]

**A**

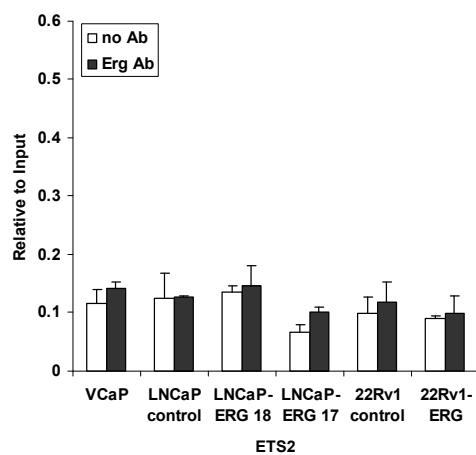

**B**

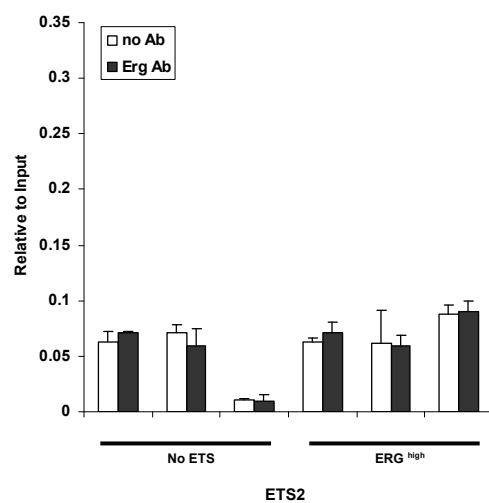

Supplement: Figure S7 — Negative control experiments for ChIP assays in ERG expressing and non-expressing cell lines and in ERGhigh and NoETS tumors. (0.04 MB PDF) [file pone.0010547.s011.pdf]

**A**

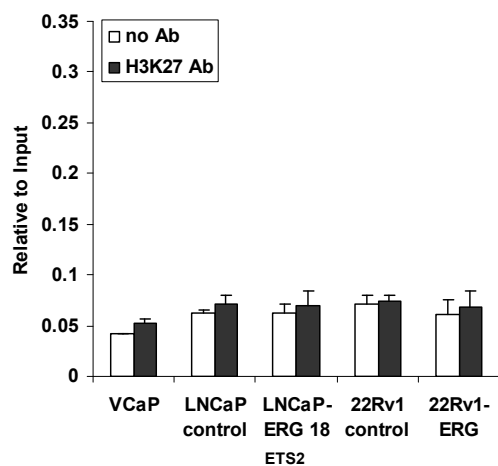

**B**

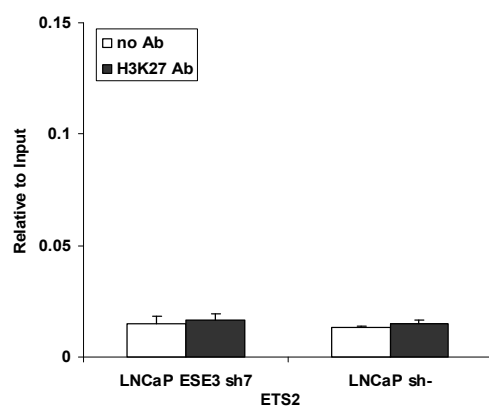

Supplement: Figure S8 — Negative control experiments for ChIP assays in parental and ERG-expressing LNCaP cells and parental and ESE-kd LNCaP cells. (0.04 MB PDF) [file pone.0010547.s012.pdf]

**A**

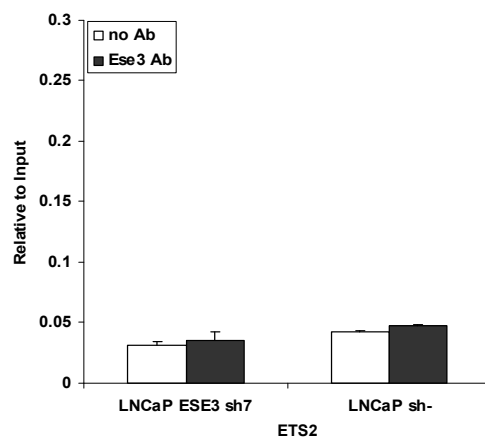

**B**

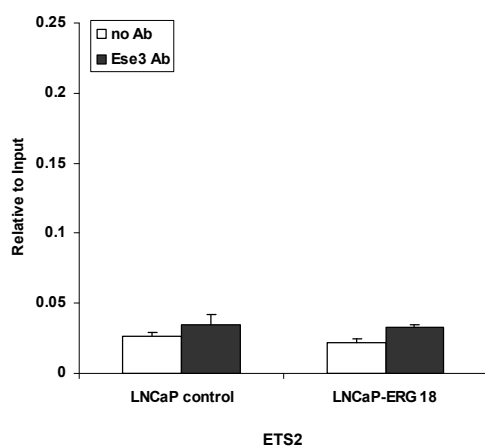

Supplement: Figure S9 — Negative control experiments for ChIP assays in parental and ESE-kd LNCaP cells and parental and ERG-expressing LNCaP cells. (0.04 MB PDF) [file pone.0010547.s013.pdf]
